# Post stroke depression: pathogenesis and molecular mechanisms of natural product-based interventions

**DOI:** 10.3389/fphar.2025.1595160

**Published:** 2025-05-19

**Authors:** Haonan Gao, Yanyan Sai, Ruirui Shang, Xia Zhong, Linghui Kong, Jie Liu, Kedong Wang

**Affiliations:** ^1^ First Clinical Medical College, Shandong University of Traditional Chinese Medicine, Jinan, Shandong, China; ^2^ University Town Hospital, Affiliated Hospital of Shandong University of Traditional Chinese Medicine, Jinan, Shandong, China; ^3^ College of Rehabilitation Medicine, Shandong University of Traditional Chinese Medicine, Jinan, Shandong, China; ^4^ Institute of Child and Adolescent Health, School of Public Health, Peking University, Beijing, China; ^5^ Committee of the Communist Youth League, Binzhou Medical University, Yantai, Shandong, China

**Keywords:** post stroke depression, natural products, antidepressant, pathological mechanism, molecular mechanism

## Abstract

Post-stroke depression (PSD) is a common sequela of stroke and a neuropsychiatric disorder associated with poor recovery, significant cognitive deficits, and reduced quality of life. Many natural products (NPs), known for their diverse biological activities, low toxicity, as well as multi-targeting capabilities, offer distinct advantages in PSD treatment by modulating pathological mechanisms. However, a comprehensive summary of the pathological mechanisms of PSD and the molecular mechanisms of NPs intervention is currently lacking. This review aimed to investigate the pathological mechanisms of PSD. It also explored the pharmacological mechanisms by which NPs exerted anti-PSD effects and in-depth discussion the limitations of current studies. Furthermore, it proposed novel methodologies for future preclinical and clinical translation in PSD research.

## 1 Introduction

Post-stroke depression (PSD) refers to depression occurring after a stroke (Villa et al., 2018). As the number of stroke survivors has increased in recent years, PSD has become a prevalent consequence, with approximately one-third of stroke survivors developing PSD (Castilla-Guerra et al., 2020; Hackett et al., 2005). A meta-analysis of 61 studies involving 25,488 patients reported an overall depression incidence of 31%, with the cross-sectional prevalence of PSD estimated between 18% and 33% (Mitchell et al., 2017; Ayerbe et al., 2013; Jørgensen et al., 2016). Compared with stroke patients without depression, those with PSD have a higher mortality rate (risk ratio for PSD and all-cause mortality: 1.6–1.9) (Cai et al., 2019), greater cognitive impairment (Serrano et al., 2007), lower quality of life (Hilari et al., 2012), and higher rates of suicidal ideation (Bartoli et al., 2017). This condition not only imposes a significant burden on patients and their families, but also negatively impact on the economy and society. At present, drug therapy for PSD typically start with selective serotonin reuptake inhibitors (SSRIs) such as escitalopram or SNRIs such as duloxetine or venlafaxine. If the initial therapy fails, tricyclic antidepressants (TCAs) such as nortriptyline should be considered (Tan et al., 2015; Min et al., 2016; Deng et al., 2017; Starkstein and Hayhow, 2019; Qin et al., 2018; Cui et al., 2018; Cipriani et al., 2018).

Although PSD patients generally tolerate these treatments well, clinicians must consider individual characteristics, potential drug interactions (e.g., with anticoagulants), and side effects (e.g., the anticholinergic effects of TCAs and the influence of SNRIs on blood pressure), which may significantly affect outcomes (Espárrago et al., 2015). Consequently, the development of more effective and safer antidepressant treatments have emerged as a pressing concern. Natural products (NPs), derived from plants or other biological sources, provide a promising avenue for drug development (Cui et al., 2023). They offer notable therapeutic effects, fewer adverse reactions, and potential synergistic interactions among numerous components. Compared with synthetic small-molecule drugs, NPs have advantages including abundant availability, cost-effectiveness, as well as accessibility, making them significant candidates for novel antidepressant development (Yuan et al., 2023).

The main biological factors linked to PSD include monoamine neurotransmitters, the hypothalamic-pituitary-adrenal (HPA) axis, glutamate (Glu)-mediated neuroexcitability, neural circuits, neuroplasticity, neuroimmunity, and the gut-brain axis (GBA). When these mechanisms are disrupted or impaired, PSD can develop (Li et al., 2014; Taylor et al., 2013; Noonan et al., 2013; Spalletta et al., 2006; Sun et al., 2024). Therefore, targeting these pathways is an important approach for the treatment of PSD. However, there is a lack of a comprehensive systematic review examining the connection between these biological factors, the physiopathology of depression, and the intervention of NPs in PSD.

In this review, we systematically presented an overview of monoamine neurotransmitters, the HPA axis, Glu-mediated neuroexcitability, neural circuits, neuroplasticity, neuroimmunity, and the GBA, highlighting their links to PSD and the pharmacological mechanisms by which NPs modulate these pathways to intervene in PSD. This review aimed to provide a scientific basis for future basic research and clinical translation.

## 2 Review methodology

**FIGURE 1:**
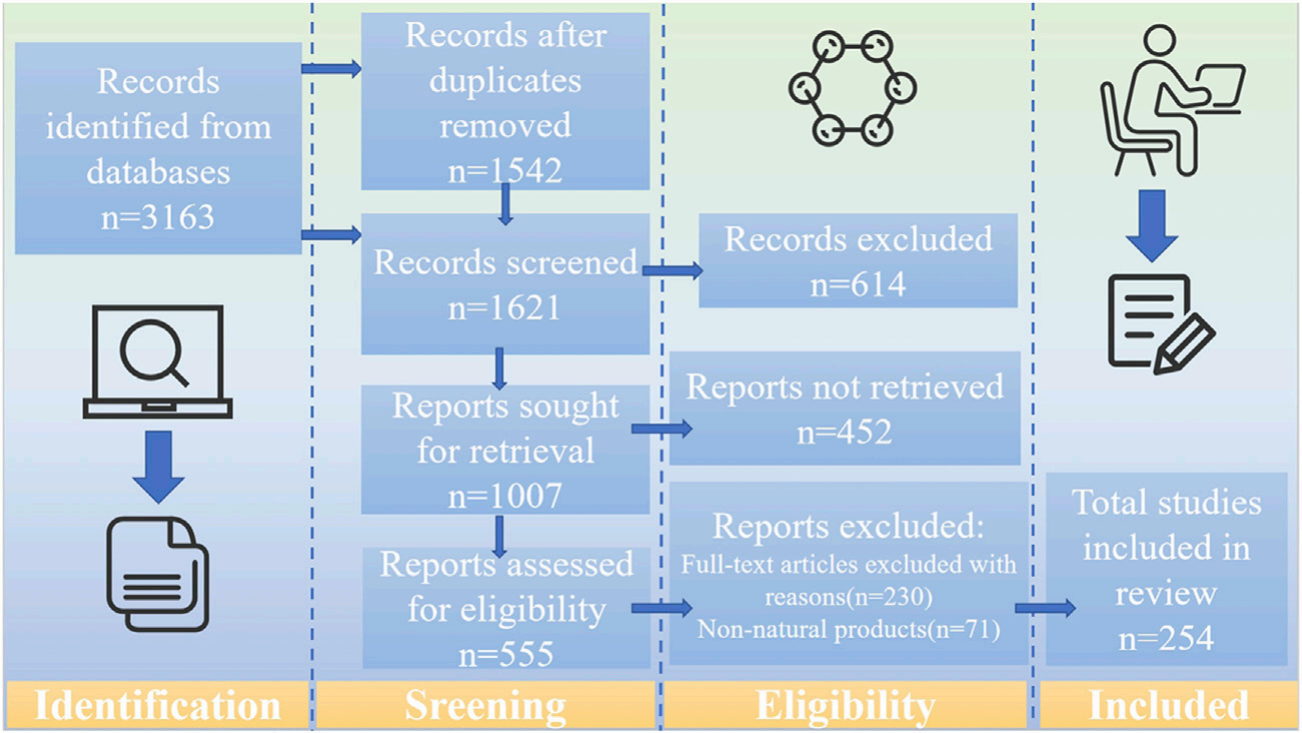
The literature search and screening flowchart.

Following a systematic evaluation based on the Preferred Reporting Items for Systematic Reviews and Meta-Analyses (PRISMA) criteria, a comprehensive review of the literature on PSD pathophysiology, NP therapies, and their toxicology and side effects was performed. Relevant studies were retrieved from three major databases, including Web of Science, PubMed, and ScienceDirect, up to February 2025. The search terms included “post-stroke depression,” “monoamine neurotransmitters,” “HPA axis,” “neurotoxicity,” “neural circuits,” “neuroplasticity,” “neuroimmunity,” “gut-brain axis,” “natural products,” “anti-post-stroke antidepressants,” and “toxicity.” The inclusion criteria were as follows: a) original publications in English, b) experimental research articles related to the pathological mechanisms of PSD, c) studies investigating the mechanisms of NPs treatment for PSD using PSD models, and d) studies related to the toxicology of NPs. The exclusion criteria were as follows: a) non-English publications, b) grey literature, conference abstracts, and case reports, c) duplicate entries, d) information on non-plant sources and crude extracts only, e) studies that did not involve NPs, such as those focusing synthetic compounds, and f) lacking of transparent methodology and objectives. Two researchers independently assessed the selected literature, verifying study reliability based on the presence of relevant keywords in the title, abstract, or full text (Figure 1).

## 3 Pathological mechanism of PSD

### 3.1 Imbalance of monoamine neurotransmitters

Monoamine neurotransmitters are essential for signal transduction in the central nervous system (CNS) and play a role in regulating movement, basal muscle tone, activity levels, emotions, attention, sleep, vascular tone, circulation, thermoregulation, and pain modulation (Ng et al., 2015). Fewer than one percent of neurons in the human brain can produce and release the neurotransmitter 5-hydroxytryptamine (5-HT, also named serotonin), a characteristic shared by all such neurons. Despite their limited number, 5-HT neurons extensively innervate the majority of brain regions and the spinal cord, contributing to a diverse array of neurological processes (Okaty et al., 2019).

Dopamine (DA) neurons regulate many important behaviors in behaviorism, including reinforcement, aversion, social reward, and responses. These functions are related to the different connectivity patterns of different subgroups of DA neurons at the molecular level. The genetic diversity, projection specificity, and functional responses of DA neurons are consistent, which may contribute to heterogeneity in the behavioral phenotypes of affective disorders and varying responses to treatment (Wu et al., 2024). Norepinephrine (NE) can directly interact with immune cells, influencing their signaling pathways and metabolic processes. In both primary and secondary lymphoid organs, NE functions as both a neurotransmitter and a neuromodulator. When sympathetic nerve terminals release NE, it immediately binds to adrenergic receptors on immune cells within these organs. Similarly, circulating adrenaline produced by the adrenal gland can interact with adrenergic receptors. Additionally, neurons express cytokine and chemokine receptors released by the immune system, facilitating the precise modulation of local immune responses (Thoppil et al., 2023). Dysregulation of monoamine neurotransmitters can have negative effects on the body.

PSD correlates with decreased concentrations of the biogenic monoamines 5-HT, DA, and NE in the CNS. The main monoaminergic nuclei are located in the brainstem, and their ascending projections extend throughout the brain, including the cortex and limbic system (Medeiros et al., 2020). During a stroke, ischemic lesions disrupt projections from the midbrain and brainstem, reducing the bioavailability of 5-HT, DA, and NE. These lesions may damage axons carrying biogenic amines from the brainstem to the cerebral cortex, leading to decreased levels of these neurotransmitters in the frontal and temporal lobes, as well as in peripheral tissues and the basal ganglia. The monoamine hypothesis suggests that depression correlates with decreased levels of monoamines, particularly 5-HT, NE, and DA (Loubinoux et al., 2012).

The pathophysiological mechanisms of PSD are multifactorial. Therefore, the establishment of an effective middle cerebral artery occlusion (MCAO) animal model or other suitable experimental animal models can facilitate the study of depression, particularly PSD (Ifergane et al., 2018; Kronenberg et al., 2014). Researchers can capture key aspects of PSD through animal models, thereby assessing behavioural performance and underlying neurobiological changes (Mușat et al., 2024).

Based on preclinical studies such as MCAO rats, it was shown that stimulation of small conductance calcium-activated K^+^ channels (SK channels) may enhance the expression of DA neurons in the ventral tegmental area (VTA) of PSD rats, resulting in behavioral changes associated with depression. In contrast, inhibition of SK channels may reduce the expression of DA neurons in the VTA, thereby alleviating depression-related behaviors. A clear association has been observed between the activation of SK channels in the VTA of PSD rats and the exacerbation of depression-related behaviors (Wang et al., 2023). Additionally, MCAO and the decrease in DA neuron density in the VTA are closely linked to a reduction in DA concentration in the brain, which is strongly associated with depression-like symptoms that can emerge during the middle stage of stroke in neonatal rats (Villa et al., 2023).

**FIGURE 2:**
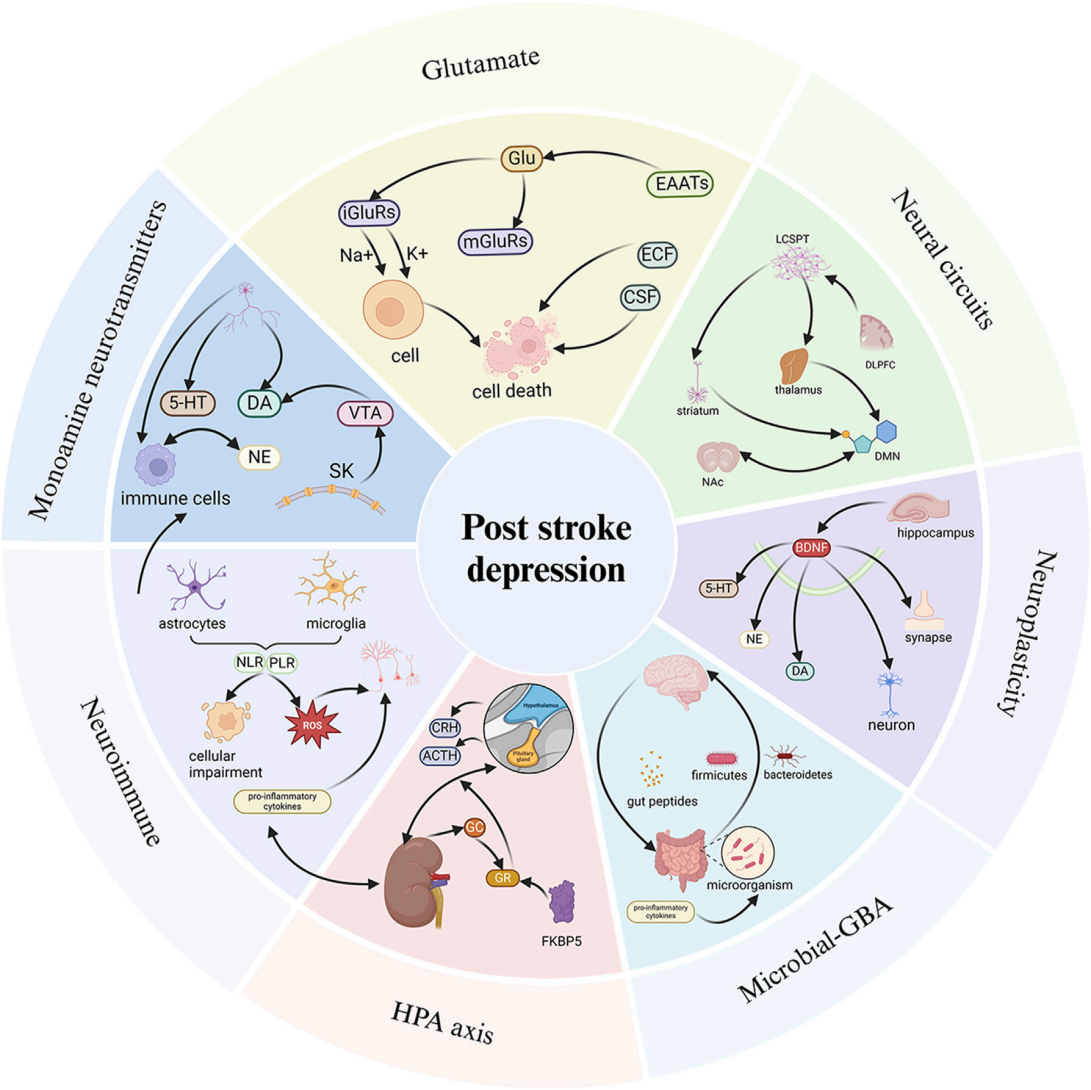
Pathological mechanisms of PSD. The pathological mechanisms of PSD involve monoamine neurotransmitters, the HPA axis, Glu-mediated neuroexcitability, neural circuits, neuroplasticity, neuroimmunity, and the GBA. When the relevant pathological mechanisms are impaired or affected, they respond through pathways that lead to the development of PSD. 5-HT, 5-hydroxytryptamine; DA, dopamine; NE, Norepinephrine; VTA, Ventral tegmental area; Glu, Glutamic acid; iGluRs, ionotropic glutamate receptors; mGluRs, metabotropic glutamate receptors; EAATS, Excitatory amino acid transporter; ECF, extracellular fluid; CSF, cerebrospinalfluid; LCSPT, limbic-cortical-striatal-pallidal-thalamic; DLPFC, dorsolateral prefrontal cortex; NAc, nucleus accumbens; DMN, default mode network; CRH, Corticotropin-releasing hormone; ACTH, adrenocorticotropic hormone; GC, glucocorticoid; GR, glucocorticoid receptors; NLR, neutrophil to lymphocyte ratio; PLR, platelet to lymphocyte ratio. Created in BioRender. Gao, H. (2025) https://BioRender.com/4ez2o5q.

Based on the regulation of neurotransmitters, PSD can be improved. The expression of 5-HT transporter genes is elevated in the hippocampus and frontal cortex following MCAO, and SSRIs have shown efficacy in the therapeutic management of PSD (Brait et al., 2022). Unilateral ischemic lesions in the medial prefrontal cortex reduce the innervation of 5-HT and NE in the affected area, as well as in other brain regions distal to the stroke. The plasticity of 5-HT axons in the forebrain projection induced by fluoxetine may help facilitate the recovery of brain injury (Zahrai et al., 2020). It can be inferred that the imbalance of monoamine neurotransmitters is an important factor contributing to PSD (Figure 2).

### 3.2 HPA axis dysfunction

Corticotropin-releasing hormone (CRH), produced and secreted by the hypothalamus, regulates the production of pituitary adrenocorticotropic hormone (ACTH), which in turn stimulates the adrenal cortex to release glucocorticoids (Werdermann et al., 2021). However, during stress events, the HPA axis becomes overactivated, leading to the release of excess glucocorticoids into the bloodstream. Numerous studies have shown that elevated levels of glucocorticoids in the hippocampus exert negative feedback regulation on the HPA axis via glucocorticoid receptors (GR), which is essential for maintaining the homeostasis of the HPA axis. The hypothalamic axis plays a key role as an adaptive system that allows the body to respond to urgent physiological and psychological challenges. Overactivity of the HPA axis has been strongly linked to the pathology of depression (Liu M. Y. et al., 2023).

The HPA axis is one of the dysregulated systems in depression, with ample evidence supporting its association with depression and other stress-related disorders. The sensitivity of GRs is primarily modulated by FK506 binding protein 5, which is linked to several mental illnesses and stress-related conditions, including severe depression, bipolar disorder, childhood trauma, post-traumatic stress disorder, as well as violent and suicidal behaviors (Menke, 2019).

CRH is one of the primary signaling peptides released after HPA axis activation during stress. As an autophagy associated protein, p62 participates in the Keap1-Nrf2 pathway through its Keap1 interaction. Recent experimental studies using the MCAO-established animal model of PSD have shown that CRH induces p62 accumulation in the prefrontal cortex of PSD rats through CRHR1. CRHR1 antagonists can inhibit the Keap 1-Nrf 2-p62 pathway by attenuating oxidative stress. These antagonists alleviate depression-like behavior and prevent hippocampal synaptic loss in PSD rats by removing excess p62, making them a new target or emerging mechanism for treating PSD (Liu et al., 2024).

HPA axis hyperfunction is one of the pathological mechanisms of PSD. Studies indicate that the severity of depression is positively associated with the level of HPA axis activation (He et al., 2003). Stroke-induced HPA axis hyperfunction significantly contributes to PSD, initiating a cascade of complex events within the neuroendocrine system, including the HPA axis. The activation of the HPA axis after a stroke is primarily driven by stress and the secretion of pro-inflammatory cytokines following significant brain damage. The involvement of HPA axis hyperactivity in PSD includes the influence of elevated ACTH, the impact of glucocorticoids on specific brain structural alterations, enzyme expression, excitotoxicity, changes in intestinal permeability, and microglial activation. These factors contribute directly or indirectly to the pathological progression of depression (Zhou et al., 2022). There is a bidirectional relationship between HPA axis dysfunction and levels of inflammatory cytokines. High cortisol levels induce inflammatory responses, which in turn exacerbate HPA axis dysfunction (Li et al., 2014). Dysregulation of the HPA axis and elevated pro-inflammatory cytokine levels may impair neurogenesis in the hippocampus, reduce neural plasticity in the prefrontal cortex, and contribute to the onset and persistence of PSD (Koo et al., 2010).

The stimulation of the HPA axis increases the concentration of glucocorticoid hormones, particularly cortisol, in the bloodstream (Douglass et al., 2023). There is a risk of developing depression if high cortisol levels persist for 3 months after a stroke. Chronic stress following a stroke may enhance the adrenal gland’s sensitivity to ACTHs, thereby prolonging hypercortisolism, which has numerous negative effects. Therefore, stroke events may serve as a key trigger for the activation of HPA axis stress in patients during the later stages, and the stimulation of the HPA axis in response to stress may contribute to the onset of depression (Aström et al., 1993).

On the other hand, in the early stages following a stroke, hypercortisolism may result from changes in cortisol metabolism and/or clearance, an enhanced adrenal gland response to ACTH, or reduced efficacy of the negative feedback mechanism between cortisol and the hypothalamus (Olsson et al., 1992). The changes in the HPA axis are part of neuroendocrine alterations, and the main neuroendocrine changes after a stroke involve the activation of the HPA axis. In some individuals, significant alterations in these neuroendocrine pathways may negatively affect functional outcomes, cognition, and emotions. Specific sequelae of stroke, like depression and sleep difficulties, may be influenced by endocrine changes, which could potentially be modified if relevant neuroendocrine abnormalities are identified and addressed (El Husseini and Laskowitz, 2014).

Focal brain injury leads to excessive activation of inflammation, disrupts HPA axis control, and further increases hormone secretion, resulting in changes in the expression and properties of cortical hormone receptors in key brain structures (primarily the limbic system). This alteration modifies the regulatory mechanism of the entire negative feedback control system (Zhanina et al., 2022). Reports indicate that CRH neurons in the paraventricular nucleus possess 5-HT receptors, suggesting a potential interaction between the HPA axis and affective disorders or stress (Heisler et al., 2007). From this, it can be concluded that the regulation of HPA axis dysfunction is intricately linked to PSD, and that interventions targeting the HPA axis offer a viable strategy for addressing PSD.

### 3.3 Glu mediated neurotoxicity

Glu serves as the primary excitatory neurotransmitter in the brain, essential for maintaining proper neuronal activity. Glu plays a crucial role in perception, including learning, memory, and other behavioral functions (Pasanta et al., 2023). Glu primarily interacts with two types of receptors: ionotropic receptors and G protein-coupled metabotropic receptors. Under physiological conditions, the activation of these receptor subpopulations induces excitatory synaptic transmission and processes associated with synaptic plasticity, such as long-term potentiation/depression, which are molecular mechanisms underlying learning and memory. Upon Glu binding, the ionotropic Glu receptor facilitates fast excitatory neurotransmission through its ion channel activity, allowing Na^+^ and K^+^ cations to traverse the plasma membrane and generate action potentials. After activation, Glu is removed from the synaptic cleft by excitatory amino acid transporters, thus terminating synaptic transmission and enabling subsequent action potentials (Neves et al., 2023).

Glu is the predominant free amino acid in the brain, positioned at the intersection of many metabolic pathways. Moreover, Glu has an excitatory effect on neuronal cells, which can lead to cell death in a process known as “excitotoxicity” (Zhou and Danbolt, 2014). Excitotoxicity is crucial in the pathophysiology of several neurodegenerative disorders, including depression. The toxic effects of excitatory neurotransmitters, particularly Glu, can induce neurotoxicity, resulting in impaired neuronal function and subsequent brain damage (Armada-Moreira et al., 2020). At the same time, the increased release of Glu can induce stress and contribute to numerous neurodegenerative disorders. Li et al. suggest that disruptions in Glu neurotransmission may contribute to the development of various mental disorders and abnormalities, including major depression (Li et al., 2019).

On the other hand, stroke is associated with a significant increase in extracellular fluid and cerebrospinal fluid Glu levels. Glu diffuses and induces neuronal injury in areas adjacent to the infarcted tissue. Excessive Glu in the brain’s extracellular fluid can activate Glu receptors, resulting in cellular swelling, apoptosis, and neuronal death. Plasma Glu levels are positively correlated with the severity of depressive symptoms in individuals with severe depression (Frank et al., 2019). Elevated levels of Glu have been found in the serum of PSD patients (Cheng et al., 2014). The association between glutamate excitotoxicity and PSD is correlative, but it needs to be further verified in subsequent experiments. In preclinical experiments, valproate (VPA) or lamotrigine (LTG), two drugs that prevent glutamate-mediated excitotoxicity by increasing inhibitory inputs or blocking sodium channels, were found to correct depressive-like behaviour to some extent with early treatment (Li et al., 2020).

Due to the widespread diffusion of Glu beyond the infarcted tissue, this increase can cause neuronal damage. Feng et al. found that the size of the infarction is a crucial factor determining the severity of PSD (Feng et al., 2014). Therefore, an elevation in Glu may impair blood flow to necrotic cerebral tissue (cerebral infarction), leading to extensive damage. The overproduction of Glu activates its receptors, resulting in cellular swelling and initiating neuronal apoptosis, eventually leading to cell death through the activation of calcium-dependent enzymes (Gruenbaum et al., 2020).

Dysregulation of the Glu system is key for the neurobiology of depression. Studies show the presence of Glu receptor proteins in the prefrontal and temporal cortices of individuals with PSD,indicating glutamatergic dysfunction (Khoodoruth et al., 2022). Substantial evidence suggest that disruptions in Glu signaling can easily lead to emotional disorders, including PSD.

Oxidative stress significantly contributes to the pathogenic processes of several types of depression, including PSD. Extensive activation of Glu receptors triggers excitotoxicity, leading to cerebral imbalance and elevated production of free radicals to toxic levels. Free radicals, which are highly reactive atoms and molecules, can damage proteins and other cellular structures (Rama et al., 2016). In addition, they also trigger mitochondrial dysfunction and neuronal damage, exacerbating excitotoxic cell death in the brain after stroke. Some free radicals (such as superoxide/hydroxyl radicals), are generated through excitotoxicity. The degeneration of neurons impairs the transmission of sensory information, including emotional regulation (Naghavi et al., 2019), which is linked to severe mental disorders like PSD after a stroke. Therefore, preventing and regulating Glu-mediated neurotoxicity is crucial for both the prevention and treatment of PSD.

### 3.4 Specific changes in neural circuits

The neural circuit is a structured system capable of transmitting and process incoming information. These circuits are also adaptable, capable of substantial enhancement and reorganization in response to development, environmental stimuli, pain, or injury (Walter et al., 2023). The dynamic remodeling of neural circuits is an ongoing process that influences neuronal activity, and disruption of this process can lead to mental illnesses. In the human brain, neurons are interconnected by billions of synapses within the CNS. The brain’s functionality depends on the exchange and transfer of information between these synapses, with the resulting neural activity providing excitation, inhibition, or regulatory signals. The pathways that transmit information between neurons are known as neural circuits, which create intricate networks within various brain regions. Any alteration in these paths can serve as a sensitive indicator of the changes in the neural circuit (Xiong et al., 2023).

Resting-state functional connectivity (FC) is useful for evaluating synchronous patterns of spontaneous neuronal activity and offers insights into the interconnections across areas within neural circuits or networks (Zhong et al., 2018). Research utilizing resting-state FC has shown that Parkinson’s disease (PD) is associated with alterations in various networks, including the default mode network (DMN) (posterior cingulate cortex seeds), emotional networks (anterior cingulate cortex [ACC] seeds), and cognitive control networks (dorsolateral prefrontal cortex seeds) (Balaev et al., 2018; Shi et al., 2017a; Vicentini et al., 2017; Zhang P. et al., 2018). The selected seeds for functional connectivity analysis mostly pertain to the disruption of severe depression, overlooking the impact of localized brain damage as the principal cause of PSD. The lesion site of PSD is commonly associated with the depression circuitry, particularly the left dorsolateral prefrontal cortex (DLPFC). Depressed individuals show a significantly stronger correlation with these lesions compared to non-depressed individuals. After a stroke, PSD patients demonstrate specific changes in their depression circuitry, including increased connectivity between the bilateral lingual gyrus and superior frontal gyrus, as well as between the contralateral middle frontal gyrus and ACC with the DLPFC (Fan et al., 2023).

The limbic-cortical-striatal-pallidal-thalamic (LCSPT) circuit is closely related to emotion regulation (Sheline, 2003). This circuit originates from the DLPFC, projects to the new striatum and thalamus, and then loops back to the cortex, primarily involving the limbic system, cortex, striatum, white matter, and thalamus (Tekin and Cummings, 2002). The proper functioning of the LCSPT circuit requires the integrity of white matter fibers in the brain. Disruption of white matter integrity impairs the LCSPT circuit, potentially leading to emotional disorders, such as depression. Microstructural damage to the white matter in regions like the frontal lobe (particularly the DLPFC), temporal lobe, and knee of the corpus callosum may contribute to the onset of PSD, with the severity of white matter damage correlating to the intensity of PSD (Liang et al., 2021).

PSD is characterized by reduced mutual inhibition between the functional circuits of the nucleus accumbens (NAc) and the DMN, along with changes in volume and microstructure within these networks. Consequently, the atypical network dynamics observed in individuals with PSD may be influenced by subsequent and extensive alterations in gray and white matter, even in regions distant from the lesion site. The disrupted link between reward processing and the DMN provides a plausible explanation for the development of post-stroke depressive symptoms. Alterations in blood oxygen level dependent signals suggest that the NAc is functionally linked to various subcortical structures, as well as medial frontal and temporal cortical areas. Similarly, the DMN encompasses a widespread regional network across the frontal and parietal cortices. Structural lesions in different areas may disrupt functional connections both within and across these networks (Oestreich et al., 2022).

The ACC is a key structure associated with emotional processing. As part of the brain’s limbic system, the ACC integrates with the limbic cortex (Passarotti et al., 2010) and plays a crucial role in emotional networks, including the DMN and attention networks. It has extensive neuronal connections to the amygdala, thalamus, and hippocampus (Enzi et al., 2012). When used as a seed for connectivity analysis, the ACC exhibits a significant reduction in FC with the prefrontal cortex, cingulate cortex, and motor cortex, alongside an increase in connectivity with the hippocampal gyrus, parahippocampal gyrus, insula, and amygdala. The stroke-affected area reduces excitability in the ipsilateral brain region, impacting emotional regulation through the prefrontal-limbic circuit and contributing to the onset of depression (Shi et al., 2017b). These findings suggest that varying degrees of neural circuit damage occur in PSD patients, and maintaining the stability of neural circuits is an effective way to improve PSD.

### 3.5 Impaired neuroplasticity

Neuroplasticity, often referred to as “brain plasticity,” is characterized as any alteration in the neural system that occurs throughout an individual’s lifespan. This means the nervous system can undergo changes in various ways. Neuroplasticity refers to the alterations in the nervous system induced by experience, including its ability to adapt to internal or external stimuli via the reorganization of its structure, function, and connections (Axelrod et al., 2023). Neuroplasticity is interconnected in several ways and can be seen as a continuous cycle of human activity that modifies brain connections and influences behavior (Wallace et al., 2023). It is key to understanding brain development, learning, and the regulation of CNS homeostasis. Broadly speaking, “neuroplasticity” refers to the ability of neural tissue to undergo changes in both normal function and pathological processes. The mechanisms of neuronal plasticity include the regulation of synaptic strength (i.e., synaptic plasticity), structural remodeling, as well as the regulation of intrinsic characteristics of neurons, like excitability or firing rate (Dzyubenko and Hermann, 2023).

The changes in neurotrophic factors lay the basis for impaired neural plasticity, which is directly linked to the onset and progression of depression. Antidepressant therapies are believed to exert their beneficial effects by enhancing the nutritional signals that promote neuronal and synaptic plasticity (Levy et al., 2018). Neurotrophic growth factors and their associated signaling pathways are primary contributors to brain plasticity, and dysfunction in these pathways is often correlated with depressive symptoms (Pittenger and Duman, 2008). Brain-derived neurotrophic factor (BDNF) is a key neurotrophic factor that plays a crucial role in neuronal development, differentiation, and survival. It is also involved in the regulation of neurogenesis and synaptic plasticity (Lu et al., 2014).

Growth factors and associated neurotrophic signals are essential for the growth and maintenance of the CNS (Greenberg et al., 2009). Abnormal nutritional support in the cortical regions that regulate emotions and mood may contribute to the pathophysiology of depression (Krishnan and Nestler, 2008). In addition, antidepressants require brain plasticity pathways to correct impairments in neuronal and synaptic plasticity, which are often linked to emotional disorders (Pittenger and Duman, 2008).

The concept of neuroplasticity suggests that the depletion of BDNF significantly contributes to the pathophysiology of PSD, and its restoration may be a crucial mechanism behind the effectiveness of antidepressant medications. BDNF, a member of the neurotrophic factor family, is expressed in both the central and peripheral nervous systems, with the highest expression observed in the cortex and hippocampus. Clinical studies indicate that variations in blood BDNF levels may serve as an early marker of depression in stroke patients. However, the use of BDNF as a marker still needs to be further refined in subsequent studies. Reduced BDNF levels in the prefrontal cortex and hippocampus correlate with the onset of PSD, and elevating BDNF levels in the prefrontal cortex may serve as a crucial intervention for treating PSD (Chen et al., 2020).

BDNF serves as a neurotrophic agent for DA neurons in the substantia nigra, promoting the survival of cultured human and rat midbrain dopaminergic neurons. Preclinical studies have shown that long-term infusion of BDNF into the midbrain of rats increases levels of BDNF in several regions, including the neocortex. Additionally, it enhances the turnover rate of 5-HT and the level of NE in many forebrain regions, such as the basal ganglia and hippocampus. Infusion of BDNF into the periaqueductal gray matter and dorsal or substantia nigra has antidepressant-like effects (Youssef et al., 2018).

Disruptions in BDNF signaling are linked to neurological diseases, such as major depression. In fact, serum BDNF levels are decreased in patients with PSD. Studies indicate that elevated BDNF protein levels in the hippocampus may mitigate behaviors linked to depression. The decreased expression of BDNF in the hippocampus, which affects neural plasticity, synaptic architecture, and function, suggests that low BDNF levels contribute to a decline in hippocampal neurogenesis, leading to PSD (Chen et al., 2015). Therefore, increasing BDNF levels to repair neural plasticity is a promising approach for treating PSD. However, upstream/downstream signaling components and their phenotypic manifestations in brain cells have not been well clarified in current BDNF research, which is mostly experimental. Furthermore, the lack of evidence linking serum BDNF levels to concentrations in the CNS limits the therapeutic significance, and further research is necessary to determine the causal linkage between peripheral and central BDNF.

### 3.6 Neuroimmune damage

Damage to the neuroimmune system can significantly impact PSD, with particular emphasis on neutrophils and neuroinflammation. Neutrophils, a type of myeloid white blood cell, are key responders to acute inflammation. They serve as the primary defense mechanism of the host against various pathogens, including bacteria, fungi, and protozoa. The localization of neutrophils at the site of infection is crucial; therefore, a decrease in neutrophil levels in the blood can lead to severe immune deficiencies in humans (Liew and Kubes, 2019).

Neuroinflammation in the brain is an innate immune response primarily mediated by reactive glial cells (astrocytes and microglia), which secrete cytokines, chemokines, reactive oxygen species (ROS), and other pro-inflammatory mediators (Sanz et al., 2024). These substances can affect neuronal activity in both the surrounding and CNSs (Li et al., 2024).

After stroke, both astrocytes and microglia undergo a series of morphological and functional changes. Due to the phenotypic transformation of astrocytes and microglia, A1/A2 and M1/M2, different subtypes of microglia and astrocytes play different roles in PSD. Astrocytes and microglia in the CNS play an important role in the pathological process of PSD by producing cytokines such as interleukin (IL), tumor necrosis factor (TNF) and interferon (IFN) (Ogłodek, 2022; Wang L. et al., 2019; Lu and Wen, 2024).

#### 3.6.1 Neutrophils

Indicators of inflammation, such as the neutrophil to lymphocyte ratio (NLR) as well as the platelet to lymphocyte ratio (PLR), are linked to both depression and stroke. Inflammation significantly contributes to acute ischemic stroke and depression by activating different pro-inflammatory markers, with evidence suggesting its involvement in the onset of PSD (Ferrari and Villa, 2017; Becker, 2016). Moreover, patients with severe depression have elevated levels of NLR and other anti-inflammatory cytokines (such as interleukin [IL]-10 and C-reactive protein) compared to healthy individuals (Demir et al., 2015; Demircan et al., 2015), and elevated NLR levels can increase the risk of suicide in depression patients (Ekinci and Ekinci, 2017).

Acute ischemic stroke has been shown to trigger an inflammatory response, as evidenced by significantly elevated plasma levels of pro-inflammatory cytokines and other inflammatory indicators. Pro-inflammatory cytokines may initiate inflammatory cascade responses (Waisman et al., 2015), and the production of cytokines linked to inflammation is strongly correlated with worse prognosis and larger infarct size in stroke patients (Bodhankar et al., 2014). The NLR and PLR have become recognized biomarkers for assessing overall inflammatory status. In individuals with depression, elevated levels of NLR and PLR are associated with increased oxidative stress and cytokine production (Kasama et al., 2005). NLR is also linked to ischemic stroke and poorer prognosis (Goyal et al., 2018).

Research has found that patients with severe depression who have not received antidepressant treatment show elevated NLR. However, after 3 months of therapy with SSRIs, the NLR levels return to normal (Demircan et al., 2015). Upon reaching the injury site, elevated concentrations of pro-inflammatory cytokines and inflammatory biomarkers correlate with the peak recruitment and activation of neutrophils (Cirée et al., 2004). The secretion of these inflammatory cytokines exacerbates the immune response, resulting in cellular impairment and the generation of ROS. Inflammation alters cerebral neuroendocrine function, reducing the synthesis and release of monoamine neurotransmitters, which contributes to the onset of PSD (Celikbilek et al., 2013). It can be inferred that the increase in neutrophils plays a significant role in the development of PSD.

#### 3.6.2 Neuroinflammation

Neuroinflammation and other cytokines are believed to be associated with the causes of depression (Bauer and Teixeira, 2019; Shariq et al., 2018). The inflammatory response involving immune-active glial cells, along with pro-inflammatory cytokines and chemokines, plays a critical role in both ischemic and hemorrhagic strokes (Infantino et al., 2022).

In the early stages, M2 microglia release anti-inflammatory factors to repair damaged neurons, and A2 astrocytes provide neuroprotection against cerebral ischaemic injury. If the injury persists, microglia shift to a pro-inflammatory state, secreting factors that not only amplify inflammation but also exacerbate neuronal damage. Inflammatory mediators and reactive substances released by activated M1 microglia can disrupt astrocyte function, reduce neurotrophic support, and impede hippocampal neurogenesis (Kreisel et al., 2014). Ischaemic stimuli and microglia-mediated inflammatory responses trigger astrocyte activation, and IL-1β, IL-6, IL-18 and TNF-α secreted by A1 astrocytes exacerbate neuroinflammation and worsen brain tissue damage (Ponath et al., 2017; Lian et al., 2015).

Inflammation is a consequence of ischemic injury and plays a dual role after a stroke. Initially, it promotes the clearance of necrotic tissue, laying the foundation for the repair and reconstruction of affected brain regions. However, this inflammatory response can also negatively impact stroke prognosis by exacerbating tissue damage and promoting the progression of ischemic brain injury (Candelario-Jalil et al., 2022). After a stroke, ischemic neurons release a series of mediators, including cytokines, chemokines, adhesion molecules, and ROS, due to impaired blood flow, activation of white blood cells in blood vessels, and the release of pro-inflammatory agents from ischemic endothelium and brain parenchyma. These inflammatory mediators further exacerbate tissue damage and accelerate the development of ischemic brain injury (Feng et al., 2024).

In a rat model, stimulation of the cerebellar fastigial nucleus downregulated TNFRSF1A expression by upregulating miR-29c expression, thereby inhibiting the expression of inflammatory cytokines and reducing the severity of PSD. This further confirms the close relationship between neuroinflammation and PSD (Wang M. et al., 2019).

**FIGURE 3:**
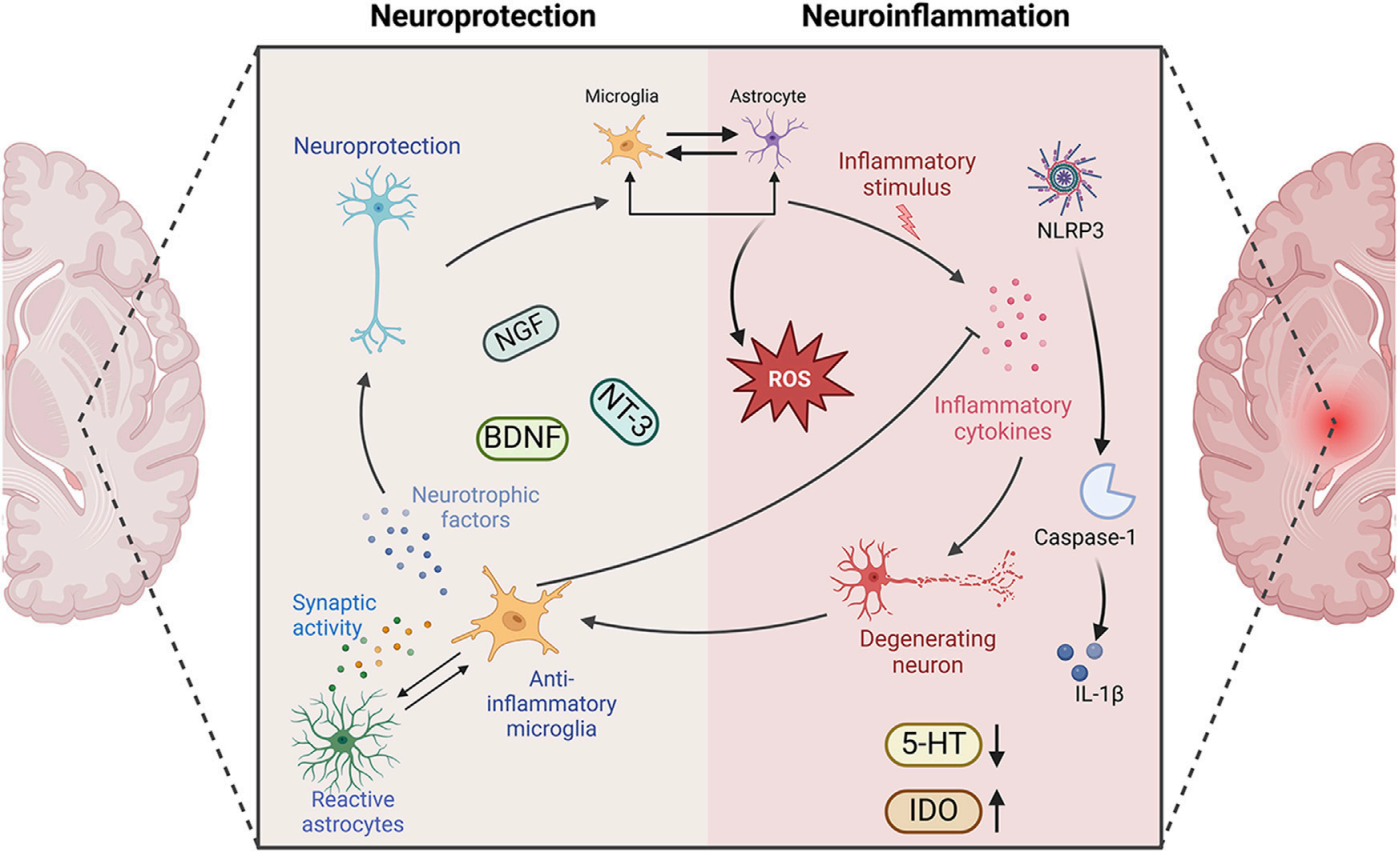
Neuroprotection versus neuroinflammation in PSD. Neuroinflammation and PSD are closely related. The immune-active glial cells, represented by astrocytes and microglia, play different roles in PSD. They play anti-inflammatory, pro-inflammatory, and corresponding neuroprotective roles in the development of neuroinflammation. NGF, nerve growth factor; BDNF, brain derived neurotrophic factor; NT-3, neurotrophins-3; 5-HT, 5-hydroxytryptamine; IDO, indoleamine2,3-dioxygenase; IL-1β, Interleukin-1β; Caspase-1, cysteinyl aspartate specific proteinase-1; NLRP3, NOD-like receptor family pyrin domain containing 3. Created in BioRender. Gao, H. (2025) https://BioRender.com/r56rbs3.

Inflammation plays a crucial role in the repair process of secondary brain injury and stroke. Following an acute stroke, both central and peripheral immune-inflammatory responses are immediately activated. Pro-inflammatory cytokines then trigger and amplify the inflammatory response, leading to malfunction of the adrenergic system, overactivity of the HPA axis, widespread activation of indoleamine 2,3-dioxygenase (IDO), rapid depletion of 5-HT in regions like the left frontal and temporal cortex, and ultimately the onset of depressive episodes (Spalletta et al., 2006; Chen H. et al., 2022; Nedic et al., 2021; Troubat et al., 2021). It can be concluded that controlling the neuroinflammatory response is of great significance for improving PSD (Figure 3).

### 3.7 Microbial-GBA disorders

The gastrointestinal system interacts with the brain in a reciprocal relationship known as the GBA. The brain regulates gut function through the HPA axis and the autonomic nervous system. Conversely, the gut influences CNS function by utilizing various microbial-derived metabolites, neuroactive substances, and gut hormones, which are transmitted to the brain via the enteric nervous system, the vagus nerve, the circulatory system, or the immune system (Liu et al., 2022). Resident microbial communities in the gut are key regulators of the GBA. These communities, which include bacteria, fungi, viruses, and other living organisms, are collectively referred to as the microbiome (Davenport et al., 2017).

The microbiota may impact the CNS, particularly by fostering bidirectional interactions between the brain and gut. The brain and gut can influence each other’s activities through neuroendocrine, neuroimmune, and sensory molecular pathways mediated by the microbiota (Wilkowska et al., 2021; Lach et al., 2018). The relationship between the brain and gut microbiota (GM) may profoundly affect stress, anxiety, cognition, and neuropsychiatric conditions such as depression, bipolar disorder, schizophrenia, and anxiety (Limbana et al., 2020; Nikolova et al., 2021).

Intestinal peptides modulate endocrine function and may interact with the CNS. Research on these peptides not only pertains to dietary consumption but also to stress-related behaviors and responses to such circumstances. The composition of GM can affect the permeability of the intestinal barrier, resulting in the secretion of gut peptides that enter brain cells in different ways and exert different effects. This variation may indicate distinct roles of peptides in organisms. Such differences can lead to changes in behaviors, ranging from reduced emotional engagement to depression-like behaviors (Lach et al., 2018).

Studies in both humans and animals indicate potential changes in the GM composition between healthy individuals and those with depression. The most significant association is the Firmicutes/Bacteroidetes ratio. In preclinical animal studies, it was found that rodents with higher levels of Bacteroidetes and lower levels of Firmicutes in the gut tended to exhibit depression-like behaviours (Labus et al., 2017; Yu et al., 2017).

The composition of the GM may influence the release of gut peptides and regulate the balance of the endocrine system. Excessive secretion of pro-inflammatory cytokines can alter the GM, as these cytokines affect gut peptides that are absorbed via the permeable intestinal barrier. This imbalance in the quantity of peptides reaching the brain may serve as a catalyst for alterations in the microbiota, resulting in behaviors resembling depression (Młynarska et al., 2022).

The changes in microbial communities within and between individuals are key drivers of stroke risk factors. An experimental analysis of the oral and fecal microbiota in African American participants from various countries revealed that individuals with cerebrovascular risk factors, like hypertension, hyperglycemia, along with hypertriglyceridemia, had high levels of oral *Streptococcus*, Prevotella, and *Klebsiella* (Fei et al., 2019). A study involving 100 patients with atherosclerosis showed that plasma levels of tryptophan, indole, indole-3-propionate, as well as indole-3-aldehyde were inversely linked with the progression of atherosclerosis (Wikoff et al., 2009). In contrast, the ratio of canine uric acid to tryptophan was positively associated with atherosclerosis (Cason et al., 2018). It suggests that the metabolic diversion of tryptophan from the indole pathway (controlled by bacteria) to the kynurenine pathway (regulated by the host) may negatively affect atherosclerosis, a significant risk factor of stroke. These findings indicate that the GM may be involved in the development of significant risk factors of stroke (Honarpisheh et al., 2022).

In animal experiments, comparisons of the GM and fecal metabolomics between PSD rats, control rats, and stroke rats revealed that compared to the control and stroke groups, the microbial phenotype of PSD rats was different. Moreover, changes in the operational taxonomic units of GM were strongly correlated with a variety of metabolites. In addition, the alterations in the gut flora of PSD rats were closely correlate with their behavioral expressions. Enrichment analysis identified essential metabolic pathways linked to PSD. These findings indicate that the GM may play a role in the onset of PSD, with its mechanism potentially involving the regulation of lipid metabolism (Jiang et al., 2021).

**FIGURE 4:**
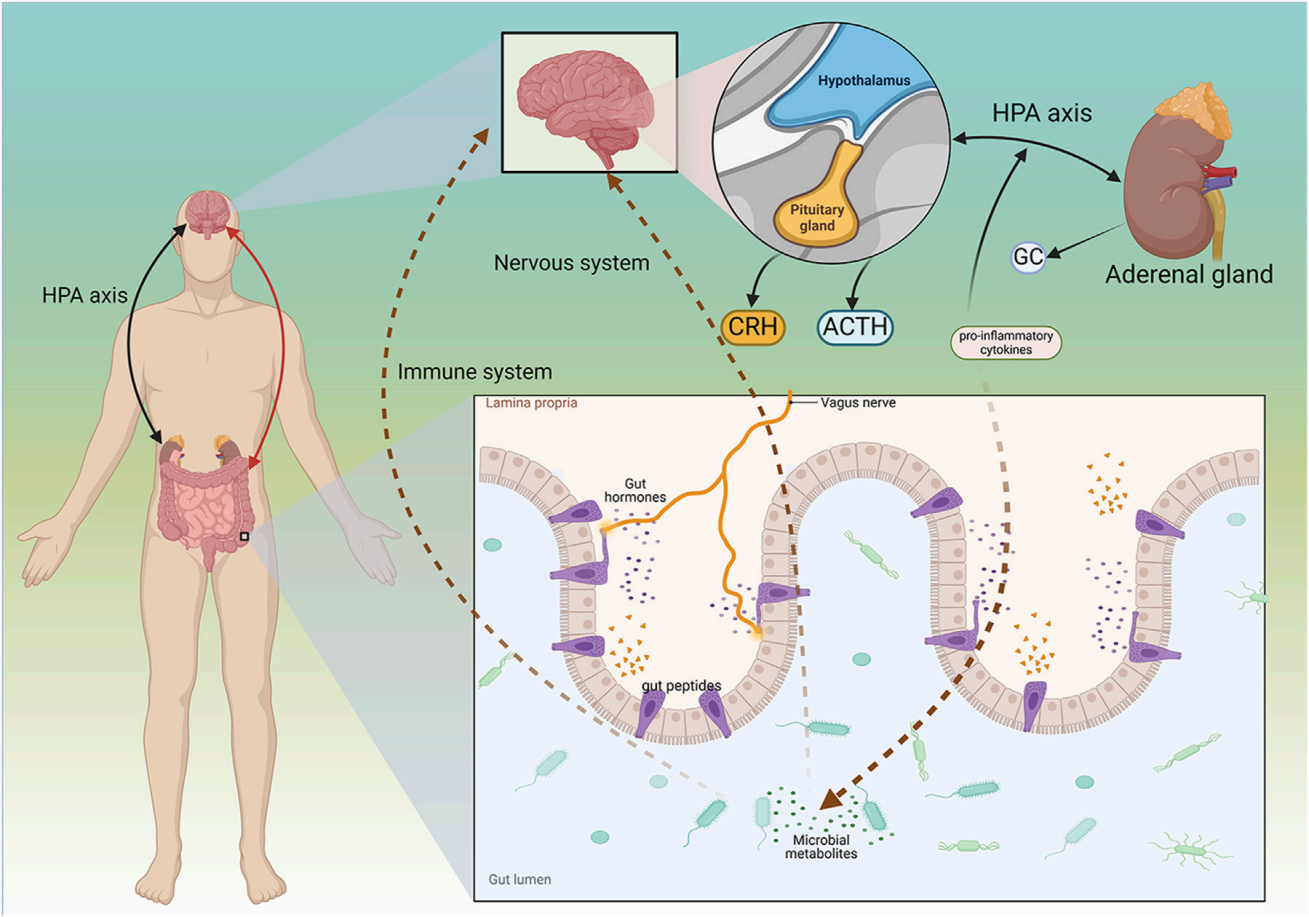
Mechanism of Microbial-GBA in PSD. The gut and the brain can interact. The brain regulates the gut through the HPA axis, and the gut regulates nervous system function through various microbes. The interaction between the brain and the gut can affect diseases such as PSD. CRH, Corticotropin-releasing hormone; ACTH, adrenocorticotropic hormone; GC, glucocorticoid. Created in BioRender. Gao, H. (2025) https://BioRender.com/e7pvowz.

In a fecal sample testing experiment involving patients with acute ischemic stroke, the genus *Streptococcus* was significantly increased in PSD patients. This includes taxonomic groups with relatively higher abundance, including strains with inflammatory characteristics, suggesting the presence of potential inflammatory mediators between GM and PSD. In addition to investigating potential pathogens, researchers also observed a decrease in bacterial abundance in PSD patients, including *Shigella* flexneri, *Clostridium* butyricum, and Waldmann’s bacteria (Yao et al., 2023). An increase in pathogenic bacteria, along with a reduction in beneficial microorganisms within the GBA, may serve as risk factors for PSD (Figure 4).

## 4 Molecular mechanisms of NPs

### 4.1 Regulation of neuroplasticity

#### 4.1.1 Synaptic plasticity

Synaptic plasticity, which refers to activity-dependent changes in the strength of neural connections, has long been considered a crucial element of learning and memory. This phenomenon occurs throughout the brain, with experience-driven alterations in the strength of synaptic connections between neurons being a central area of research (Magee and Grienberger, 2020). Activity-dependent synaptic plasticity is a fundamental characteristic of the nervous system, allowing neurons to interact and modify their connections based on prior experiences. Through this process, the nervous system can adapt by reweighting synaptic strength (Appelbaum et al., 2023).

Gastrodin, an active compound derived from the rhizome of *Gastrodia elata* Bl. (Ye et al., 2018), has various pharmacological activities, including pain relief, hypnosis, memory improvement, and antidepressant effects (Liu et al., 2018; Chen et al., 2016; Wu et al., 2023; Wang X. et al., 2021). Cannabinoid receptor 1 (CB1R) is one of the most prevalent G-protein coupled receptors (GPCRs) in the brain and plays a crucial role in synaptic and behavioral functions (Busquets-Garcia et al., 2018; Piomelli, 2025; Zou and Kumar, 2018). CB1R is highly expressed in different brain structures, cells, and subcellular locations, with its activity and expression levels directly influencing synaptic activity and behavior (Covelo et al., 2021). CB1R is a potential target for gastrodin to exhibit its neuroprotective properties. Subsequently, PKA/RhoA-related signaling pathways are activated to modulate synaptic transmission in the hippocampus, indicating an interaction between CB1R and gastrodin-related signaling pathways. Research has shown that synaptic dysfunction in hippocampal neurons may contribute to PSD. Depressive-like behavior in animals after a stroke correlates with decreased expression of CB1R on the membrane of hippocampus neurons. Effective intervention with gastrodin can reverse depressive-like behavior and synaptic dysfunction. Treatment with gastrodin may stimulate the CB1R/PKA/RhoA signaling pathway, correcting impaired synaptic formation in PSD, enhancing neurotransmission and synaptic plasticity, and demonstrating its anti-PSD effects (Wang et al., 2025).

Carnosic acid, a naturally occurring catechol-type polyphenolic diterpenoid compound found in *Rosmarinus officinalis* L., has the ability to cross the blood-brain barrier (BBB) and may contribute to neuronal regulation (Satoh et al., 2008; Wang et al., 2012). The neurotrophic factor theory posits that restoring growth factors can alleviate brain dysfunction and enhance neuronal plasticity. BDNF, which is implicated in synaptic function, has been shown to be diminished in the hippocampus and prefrontal cortex in various conditions (Cattaneo et al., 2015). Fibroblast growth factor 9 (FGF9), a member of the fibroblast growth factor family, is widely expressed in the CNS (He et al., 2024). FGF9 functions as a neuroglial activating factor, primarily expressed in the nervous system and involved in various biological processes (Lin et al., 2009). *In vivo* studies have shown that Carnosic acid tends to reduce FGF9 expression in PSD rats, while also reversing the relationship between FGF9 and its receptor FGFR-3. Furthermore, Carnosic acid has been shown to reduce infarct volume in PSD rats (Azhar et al., 2021).

Curcumin, a principal bioactive polyphenolic compound derived from the rhizomes of *Curcuma longa* L., exhibits various biochemical activities, including anti-inflammatory, antiviral, antibacterial, antioxidant, and anticancer properties. Its potent antioxidant capabilities protect cells from protein carbonylation, lipid peroxidation, and mitochondrial permeability transition (Farkhondeh et al., 2019; Amalraj et al., 2016; Kocaadam and Şanlier, 2017). In the CNS, BDNF plays crucial roles in synaptic plasticity, neuronal growth, and neuronal survival (Guo et al., 2014). Curcumin promotes the development of long non-coding RNA growth arrest-specific transcript 5 (GAS5), which activates the BDNF/TrkB signaling pathway and enhances the production of synaptic-associated proteins. By upregulating GAS5, curcumin reduces miR-10b, thereby affecting BDNF mRNA levels. In Curcumin activates the BDNF/TrkB signaling pathway in PSD rats, increasing the transcription level of BDNF. Numerous studies have shown that cyclic AMP response element-binding protein (CREB) is activated by the PI3K/Akt pathway, further promoting BDNF transcription. Given the important role of synapsin 1 (SYN1) in neurogenesis and synaptic development, animal experiments in rats indicate that curcumin effectively alleviates PSD by enhancing the expression of SYN1 (Cai et al., 2020) (Table 1).

**TABLE 1:** Modulation of PSD by natural products.

Natural product	Chemical structure	Molecular formula	Main sources	Research object and modeling method	Dosage	Route of administration	Main indicators	Molecular mechanism	References
Gastrodin	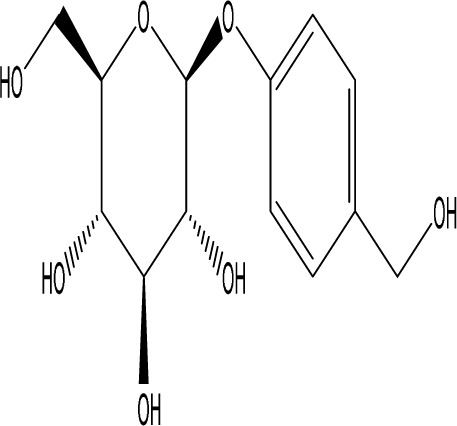	C_13_H_18_O_7_	*Gastrodia elata* Bl.	C57BL/6 mice, MCAO model	50 mg/kg, 100 mg/kg, 200 mg/kg	Intravenous administration	p-PKA↑, p(ser188)-RhoA↑, CB1R↑	Treatment with gastrodin may stimulate the CB1R/PKA/RhoA signaling pathway, hence correcting impaired synaptic formation in PSD, further enhancing neurotransmission and synaptic plasticity, and exhibiting its anti-PSD effects.	Wang et al. (2025)
Carnosic acid	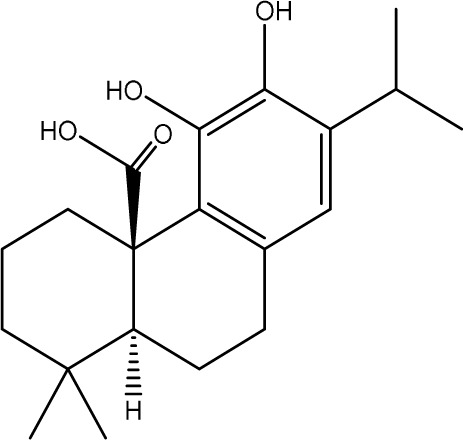	C_20_H_28_O_4_	*Rosmarinus officinalis* L.	Sprague Dawley rats, MCAO model	20 mg/kg, 40 mg/kg	Oral administration	FGF9↓, FGFR-3↑	Carnosic acid has a tendency to reduce FGF9 expression in post-stroke depression rats, while also reversing the relationship between FGF9 and FGFR-3, and reducing infarct volume in PSD rats.	Azhar et al. (2021)
Curcumin	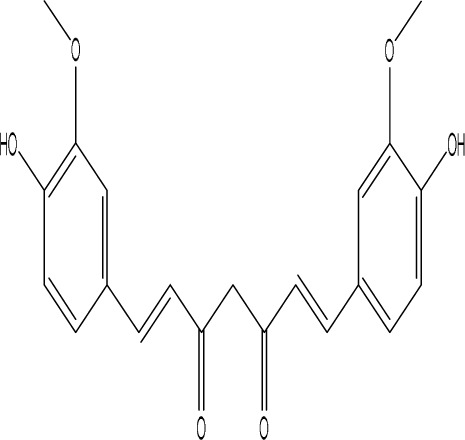	C_21_H_20_O_6_	*Curcuma longa* L.	Sprague Dawley rats, MCAO model	200 mg/kg/day	Intragastric administration	5-HIAA↓, DA↓, GAS5↑, miR-10b↓, BDNF↑	Due to the important role of SYN1 in neurogenesis and synaptic development, further animal experiments in rats have shown that curcumin can effectively alleviate PSD by enhancing the expression of SYN1	Cai et al. (2020)
Echinacoside	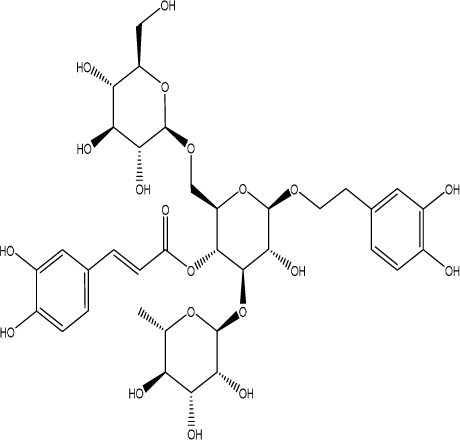	C_35_H_46_O_20_	*Cistanche deserticola* Ma; *Echinacea purpurea* (L.) Moench; *Rehmannia glutinosa* (Gaertn.) Libosch. ex Fisch. & C. A. Mey.	Sprague Dawley rats, MCAO model	7.5 mg/kg, 15 mg/kg, 30 mg/kg	Intraperitoneal injection	Nrf2↑, BDNF↑	Echinacoside enhances Nrf2 activity by facilitating its acetylation, which in turn increases the transcriptional activity of BDNF. This then triggers the BDNF/TrkB signaling pathway, which aids in the amelioration of PSD symptoms in rats.	Yang et al. (2024)
Paeoniflorin	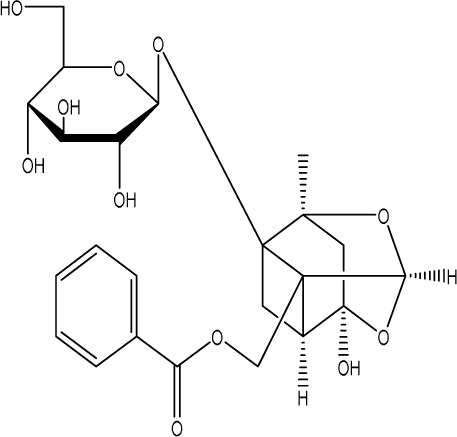	C_23_H_28_O_11_	*Paeonia lactiflora* Pall.	SpragueDawley rats, MCAO model	5 mg/kg/day	Intraperitoneal injection	BDNF↑, p-CREB↑	Paeoniflorin enhances the production of BDNF and p-CREB, thereby facilitating positive neural and mood regulation.	Hu et al., (2019)
Aloe-Emodin	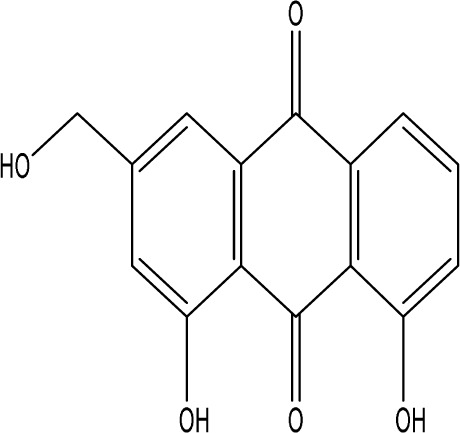	C_15_H_10_O_5_	*Aloe vera* (L.) Burm. f. or *Rheum palmatum* L.	SpragueDawley rats, MCAO model	100 mg/kg	Intragastric administration	neuron↑, BDNF↑, NTF3↑, AQP3↓, AQP4↓, AQP5↓, GFAP↓, TRPV4↓	Aloe-Emodin has positive effects on PSD rats by improving depression, significantly improving behavioural functioning in PSD rats, decreasing the level of depression, increasing their activity and curiosity, ameliorating brain tissue injuries and lesions, increasing the number of neurons in the brain tissues and reducing the number of brain tissue and brain tissue AQP3, AQP4, AQP5, GFAP, TRPV4 content, and increase BDNF, NTF3 protein expression.	Liu et al. (2023b)
Sodium Tanshinone IIA Sulfonate	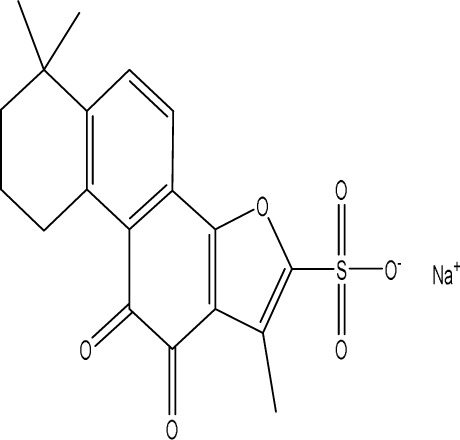	C_19_H_17_NaO_6_S	*Salvia miltiorrhiza* Bunge	SpragueDawley rats, MCAO model	8.4 mg/kg	Intravenous injection	Alb↓, mTOR↑, Dync1h1↓, Stxbp1↓, Cltc↑, Sptan1↓, PI3K/AKT↑	Analysis of signaling pathways indicated that the therapeutic mechanism of STS may engage the phosphatidylinositol signaling system, the PI3K-Akt signaling pathway, and the HIF-1 signaling pathway; additionally, enrichment results suggested that STS may be beneficial in treating sleep disorders and depression post-stroke.	Wang et al. (2022)
Saikosaponin A	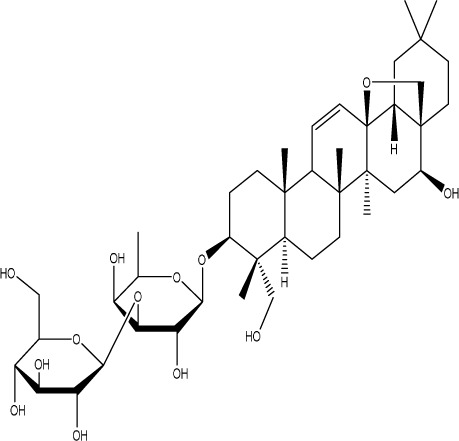	C_42_H_68_O_13_	*Bupleuri Radix* (*Bupleurum chinense* DC.)	SpragueDawley rats, MCAO model	5 mg/kg	Intraperitoneal injection	BDNF↑, p-CREB↑, Bcl-2↑, Bax↓, Caspase-3↓	SSA therapy markedly enhanced the expression of p-CREB and BDNF, decreased the expression of Bax and Caspase-3, elevated the expression of Bcl-2, and inhibited the death of hippocampal neurons, therefore ameliorating depression-like behavior in PSD rats.	Wang A. R. et al. (2021)
Morroniside	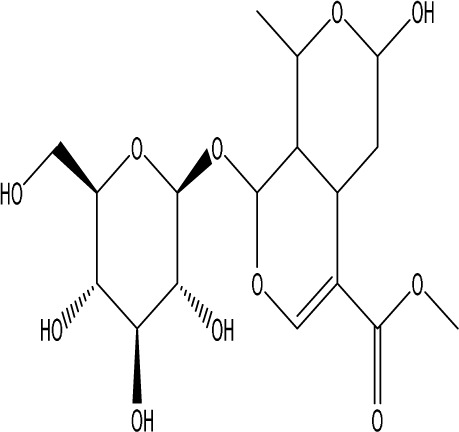	C_17_H_26_O_11_	*Cornus officinalis* Siebold & Zucc.	C57BL/6 mice, MCAO model	10 mg/kg, 20 mg/kg, 40 mg/kg	Intragastric administration	BDNF/TrkB↑, MiR-409-3p↓	Researchers observed a reduction in neuronal apoptosis and an improvement in depressive-like behaviour, which led to the conclusion that morroniside prevents neuronal death and alleviates PSD symptoms in mice via the MiR-409-3p-mediated BDNF/TrkB signaling pathway.	Qian et al. (2024)
Curculigoside	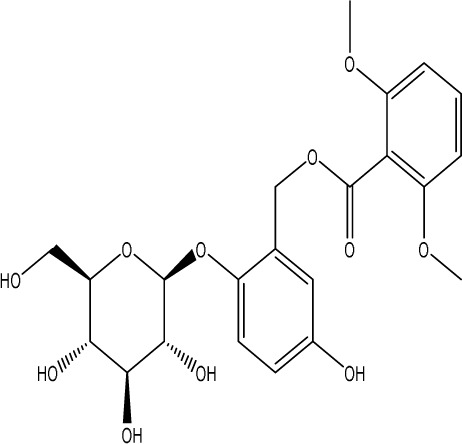	C_22_H_26_O_11_	*Curculigo orchioides* Gaertn.	SpragueDawley rats, MCAO model	10 mg/kg, 50 mg/kg	Intragastric administration	ATP↓, TFAM↓, PGC-1α↓	Curculigoside mitigates dysfunctional behaviors induced by compromised hippocampus mitochondrial oxidative phosphorylation and neurogenesis, indicating its potential in the prevention and treatment of post-stroke depression.	Zeng et al. (2024)
Ganoderic acid A	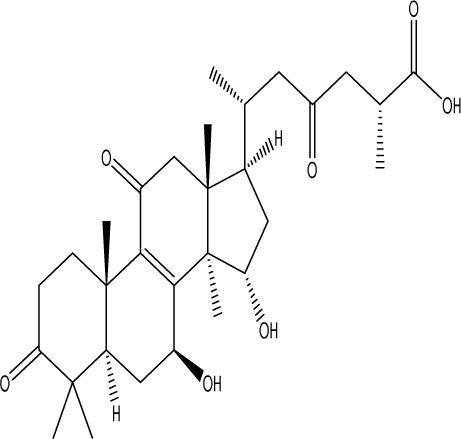	C_30_H_44_O_7_	*Ganoderma lucidum* (Curtis) P. Karst.	SpragueDawley rats, MCAO model	10 mg/mL, 20 mg/mL, 30 mg/mL	Intravenous injection	M1↓, M2↑, ERK/CREB↑	GAA has been shown to mitigate the inflammatory response in PSD rats by reducing the levels of TNF-α, IL-1β, and IL-6, while increasing IL-10, indicating an anti-inflammatory action of GAA. Additional examination of microglial cell markers suggests that the anti-inflammatory impact of GAA in PSD rats may be realized via the induction of the anti-inflammatory M2 phenotype in microglial cell polarization.	Zhang et al. (2021)
Morinda officinalis oligosaccharides	—	—	*Morinda officinalis* F. C. How	SpragueDawley rats, MCAO model	0.1 mg/g/d	Oral administration	IL-18↓, IL-1β↓, NLRP3↓	MOOs downregulated the mechanism of NLRP3 inflammatory vesicle expression in PSD rats by affecting the IκB/NF-κB p65 pathway, and MOOs inhibited the activation of microglial NLRP3 inflammatory vesicles, which attenuated hippocampal inflammation.	Li et al. (2021)
Gastrodin	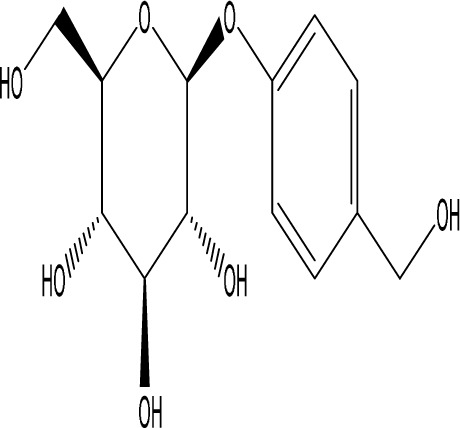	C_13_H_18_O_7_	*Gastrodia elata* Bl.	SpragueDawley rats, MCAO model	200 mg kg^−1^	Oral administration	DA↑, 5-HT↑, _L_-DOPA↑	Gastrodin has a comparable influence on the regulation of brain neurotransmitters as the pharmaceutical fluoxetine, and may serve as a potentially beneficial herbal agent for the treatment of PSD.	Zhao et al. (2019)
Huperzine A	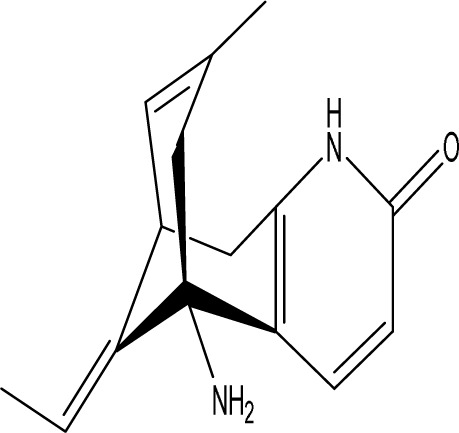	C_15_H_18_N_2_O	*Huperzia serrata* (Thunb. ex Murray) Trevis.	SpragueDawley rats, MCAO model	0.05 mg/kg, 0.15 mg/kg	Intragastric administration	5-HT_1A_R↑, p-CREB↑,BDNF↑,NE↑, DA↑, 5-HT↑	The HupA treatment markedly demonstrated antidepressant effects, enhancing neurological and cognitive functions in PSD rats, resulting in the upregulation of 5-HT_1A_R, p-CREB, and BDNF expression in the hippocampus, alongside increased levels of 5-HT in the hippocampus and prefrontal cortex, as well as elevated DA and NE levels in these regions.	Du et al. (2017)
Astragaloside VI	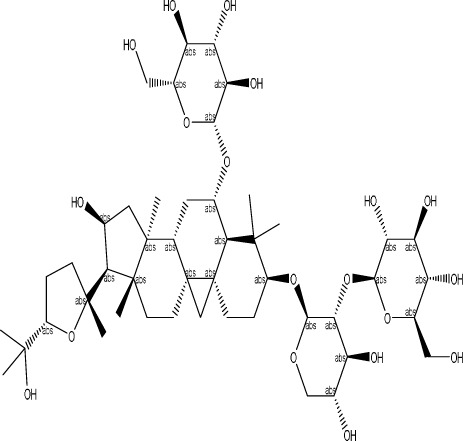	C_47_H_78_O_19_	*Astragalus membranaceus* (Fisch.)	SpragueDawley rats, MCAO model	2 μg/kg	Intravenous injection	DA↑, 5-HT↑, MEK/ERK↑	AsVI therapy markedly inhibited the reduction of dopamine and serotonin levels in the brains of PSD rats and in CORT-induced PC-12 cells. In addition, AsVI therapy enhanced the NRG-1-mediated MEK/ERK pathway, correlating with the amelioration of PSD.	Chen et al. (2022b)
Naringin	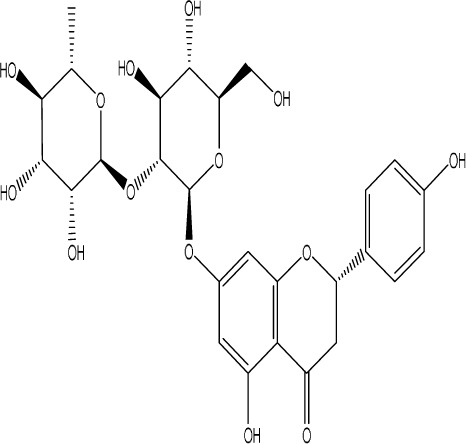	C_27_H_32_O_14_	*Citrus reticulata* Blanco	Albino mice, MCAO model	50 mg/kg, 100 mg/kg	Intraperitoneal injection	MDA↓, glutathione value↑, catalase↑, SOD↑, GST↑	Naringin pretreatment with L-NAME or 7-NI augmented its protective effect by restoring mitochondrial enzyme activities, thereby corroborating the role of nitric oxide and indicating its involvement in the protective effects of naringin against neurobehavioral, biochemical, and cellular changes in depressed mice post-stroke.	Aggarwal et al. (2010)
Rosmarinic acid	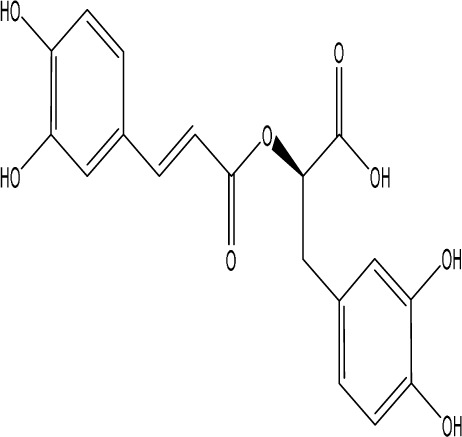	C_18_H_16_O_8_	plant groups including Labiatae, Seaweeds and Ferns	SpragueDawley rats, MCAO model	1 mg/kg, 2 mg/kg, 4 mg/kg, 8 mg/kg, 16 mg/kg, 32 mg/kg	Intraperitoneal injection	SOD↑, CAT↑, GSH↑, Nrf2↑	RA markedly reduced neurological impairments and decreased infarct volume. RA therapy mitigated depressive behaviors by diminishing the decline of SOD and CAT activities and GSH levels in the brain.	Wang et al. (2021b)
Resveratrol	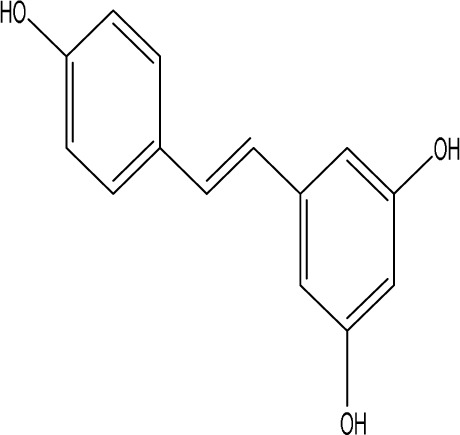	C_14_H_12_O_3_	the skins of red wine grapes, *Reynoutria japonica* Houtt., and some nuts	SpragueDawley rats, MCAO model	40 mg/kg/day	Intragastric administration	Nrf2/HO-1↑	The suppression of the Nrf2/HO-1 pathway partly mitigated the anti-inflammatory and antioxidative effects of RES, along with its enhancement of cognitive and learning abilities in PSD rats. Whereas Nrf2/HO-1 pathway activators amplify these effects.	Bai et al. (2024)
Trans-resveratrol	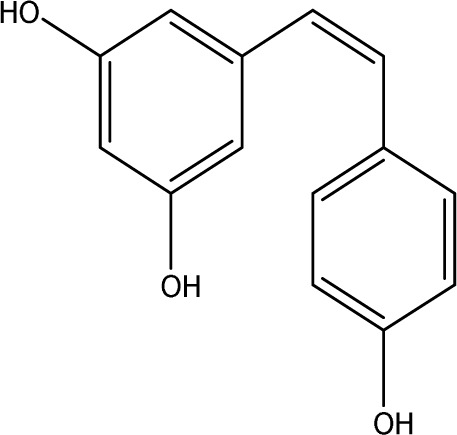	C_14_H_12_O_3_	*Reynoutria japonica* Houtt. and also in some dietary products such as grapes and red wine	SpragueDawley rats, MCAO model	10 mg/kg, 20 mg/kg, 40 mg/kg	Intragastric administration	CRF↓, BDNF↑	Trans-resveratrol enhanced BDNF expression in the frontal cortex, hippocampus, and hypothalamus of MCAO rats, indicating that BDNF expression may confer protective effects on cerebral ischemic neurons and elucidating the mechanism of trans-resveratrol’s action at the psycho-neuroendocrine level, particularly as a modulator of the HPA axis.	Pang et al. (2015)
Inulin	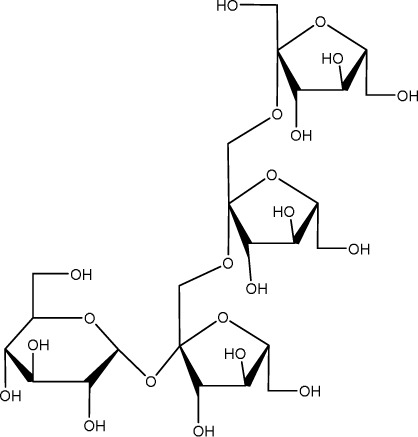	C_17_H_11_N_5_	chicory, Jerusalem artichoke, dahlia	SpragueDawley rats, MCAO model	0.3 g/L	Oral administration	mitogen-activated protein kinase↑, *Lactobacillus*↑, *Clostridium*_sensu_stricto_1↑, Ruminococcus UCG_005↓, Prevotella_9↓, Oscillospiraceae↓, Clostridia UCG_014↓	Inulin consumption elevated IGF-1 hormone levels in the body, augmented IGF-1 levels in the bloodstream, and stimulated the activation of the MAPK pathway, which alleviated post-stroke depression-like behaviour.	Shao et al. (2024)

CB1R, cannabinoid-1, receptor; PKA, protein kinase A; RhoA, Ras homolog gene family member A; FGF9, fibroblast growth factor 9; FGFR-3, fibroblast growth factor receptor 3; SYN1, Synapsin 1; Nrf2, Nuclearrespiratoty factor 2; BDNF, brain-derived neurotrophic factor; TrkB, Tyrosine Receptor Kinase B; p-CREB, phosphorylated CREB; AQP3, Aquaporin 3; AQP4, Aquaporin-4; AQP5, Aquaporin 5; GFAP, glial fibrillary acidic protein; TRPV4, transient receptor potential vanilloid 4; NTF3, neurotrophic 3; PI3K, Phosphatidylinositol 3-kinase; Akt, protein kinase B; HIF-1, hypoxia inducible factor-1; Bax, BCL2-Associated X; Caspase-3, cysteinyl aspartate specific proteinase-3; Bcl-2, B-cell lymphoma-2; TNF-α, tumor necrosis factor-α; IL-1β, Interleukin-1β; IL-6, Interleukin-6; IL-10, Interleukin-10; M2, promoted anti-inflammatory; NLRP3, nucleotide-binding oligomerization domain-like receptor protein 3; IκB, Inhibitor of NF-κB; NF-κB p65, nuclear factor kappa-B p65; 5-HT1AR, 5-hydroxytryptamine 1A receptor; CORT, corticosterone; NRG-1, Neuregulin 1; MEK, mitogen-activated extracellular signal-regulated kinase; ERK, extracellular regulated protein kinases; 7-NI, 7-Nitroindazole; SOD, superoxide dismutase; CAT, catalase; GSH, glutathione; HO-1, heme oxygenase 1; MCAO, middle cerebral artery occlusion; IGF-1, insulin-like growth factor-1; MAPK, mitogen-activated protein kinase.

#### 4.1.2 Structural plasticity

Structural plasticity occurs when pre-existing neural connections are modified via the formation of new synapses or the removal of inactive connections (synaptic pruning). Significant structural alterations associated with structural plasticity include the formation of new connections, reorganization or removal of existing ones, and changes in the complexity of neurons or networks (Bortolotto et al., 2005; Holahan and Routtenberg, 2011). Attributes of structural plasticity, such as dendritic length, branching, as well as spine density (together referred to as dendritic complexity), expand the available surface area for synaptic interactions, thereby enhancing neuronal connectivity (Matus, 2000).

Echinacoside is a phenylethanolic glycoside extracted from various plants, with multiple pharmacological features, including anti-inflammatory properties, mitochondrial protection, antioxidant capacity, anti-apoptotic actions, and neuroprotective effects in various neurological disease models (Li et al., 2022; Wu et al., 2020). Echinacoside exhibits significant antidepressant effects in the PSD model. This effect results from the stimulation of the BDNF/tropomyosin receptor kinase B (TrkB) signaling pathway, which inhibits oxidative damage and cell death while activating the antioxidant system. Nrf2 plays a key role in the activation of this signaling pathway. Echinacoside enhances Nrf2 activity by facilitating its acetylation, which, in turn, increases the transcriptional activity of BDNF. This then triggers the BDNF/TrkB signaling pathway, contributing to the amelioration of PSD symptoms in rats (Yang et al., 2024).

Paeoniflorin is a terpenoid glycoside extracted from *Paeonia lactiflora* Pall., which has been shown to possess antioxidant, neuroprotective, and antidepressant effects (Mao et al., 2012; Liang et al., 2005; Li et al., 2016). In rat animal experiments, significant antidepressant-like activity was reported in both neurological dysfunction and depression in the PSD rat model following paeoniflorin administration. In PSD rats, researchers found that paeoniflorin has neuroprotective and antidepressant properties. Paeoniflorin enhances the production of BDNF and phosphorylated CREB (p-CREB), thereby facilitating positive neural and mood regulation. Consequently, paeoniflorin exhibits antidepressant-like effects in the PSD rat model, with the mechanism likely linked to the activation of the BDNF and p-CREB signaling pathways (Hu et al., 2019).

Aloe-Emodin (AE) is a naturally occurring anthraquinone extracted from *Aloe vera* (L.) Burm. f. or *Rheum palmatum* L. (Froldi et al., 2019). It is characterized by a range of pharmacological activities, including anticancer, anti-inflammatory, antioxidant, immunosuppressive, as well as neuroprotective effects, and contributes positively to several disorders (Semwal et al., 2021). AE has beneficial effects on PSD rats by improving depression, significantly enhancing behavioral functioning, reducing depression levels, increasing activity and curiosity, ameliorating brain tissue injuries and lesions, increasing the number of neurons in brain tissues, and reducing the levels of AQP3, AQP4, AQP5, GFAP, and TRPV4 in brain tissue. Furthermore, AE increase BDNF, and neurotrophin-3 protein expression. AE may have a substantial therapeutic impact on PSD rats (Liu Y. et al., 2023).

Sodium Tanshinone IIA Sulfonate (STS) is isolated from *Salvia miltiorrhiza* Bunge and is a prospective therapeutic agent for ischemic cerebral infarction. STS protects against cerebral injury, promotes angiogenesis and neural regeneration, inhibits neuronal apoptosis, and improves ischemia-induced BBB damage (Morton et al., 2015). It has been found to have unique efficacy in clinical practice. STS can prevent progressive and sustained injury in the hemi-dark area and improve neurological deficits in MCAO/reperfusion (MCAO/R) model rats. Analysis of signaling pathways indicated that the therapeutic mechanism of STS may involve the phosphatidylinositol signaling system, the PI3K-Akt signaling pathway, and the hypoxia-inducible factor 1 signaling pathway. Additionally, enrichment results suggested that STS may be beneficial in treating sleep disorders and depression post-stroke (Wang et al., 2022).

Saikosaponin A (SSA) is a monomer of the total saponins of Saikosaponin, a triterpene saponin derived from *Bupleuri Radix* (*Bupleurum chinense* DC.), which has many pharmacological properties, including antidepressant, anti-inflammatory, antitumor, antioxidant, as well as protective effects on the brain and liver. SSA has been shown to promote neurological function and increase BDNF expression in individuals with brain damage (Jichao et al., 2017; Ye et al., 2016; Mao et al., 2016). In experimental trials using rats, SSA ameliorated depressive-like behavior and reduced hippocampal cell death after cerebral ischemia in PSD rats, possibly through the upregulation of BDNF and p-CREB. SSA therapy significantly enhanced the expression of p-CREB and BDNF, decreased the expression of Bax and Caspase-3, elevated the expression of Bcl-2, and inhibited the death of hippocampal neurons, thereby ameliorating depression-like behavior in PSD rats (Wang A. R. et al., 2021).

Morroniside is a cyclic enol ether terpene glycoside found in *Cornus officinalis* Siebold & Zucc. with significant antioxidant (Xu et al., 2006) and anti-apoptotic properties (Pi et al., 2017). Through animal experiments, researchers observed a reduction in neuronal apoptosis and an improvement in depressive-like behavior, leading to the conclusion that morroniside prevents neuronal death and alleviates PSD symptoms in mice via the MiR-409-3p-mediated BDNF/TrkB signaling pathway. The findings underscore the importance of the BDNF signaling pathway in ameliorating PSD symptoms, strongly suggesting the potential of morroniside as a therapeutic agent for PSD and offering a plausible mechanism for its treatment (Qian et al., 2024).

Curculigoside has significant permeability across the BBB. Preclinical research has shown that curculigoside exhibits various pharmacological properties, including anti-ischemia/reperfusion injury, antidepressant effects, together with protection against hippocampal damage (Yang et al., 2019; Zhao et al., 2020). Curculigoside markedly preserved hippocampal expression of doublecortin (DCX) and Nestin proteins, while increasing the number of DCX-positive and Nestin-positive cells in the dentate gyrus. The results suggest that the enhancement of mitochondrial oxidative phosphorylation with curculigoside treatment may be crucial in facilitating neurogenic repair. It may restore hippocampal neurogenesis in PSD rats by augmenting hippocampal mitochondrial oxidative phosphorylation, demonstrating significant anti-PSD potential. Curculigoside mitigates dysfunctional behaviors induced by compromised hippocampal mitochondrial oxidative phosphorylation and neurogenesis, indicating its potential in the prevention and treatment of PSD (Zeng et al., 2024).

### 4.2 Relief of neuroinflammation

#### 4.2.1 Inhibition of pro-inflammatory cytokine expression

The balance between pro-inflammatory and anti-inflammatory cytokines in the brain is a crucial determinant of neuronal function preservation by microglia. Pro-inflammatory cytokines such as IL-1, IL-3, IL-6, IL-8, IFN-γ, and TNF-α induce neuronal degeneration and neurotoxicity in brain tissues, whereas anti-inflammatory agents, including IL-1 receptor antagonists (IL-1Ra) and IL-10, function as protective components (Youn et al., 2013).

Ganoderic acid A (GAA) is a principal triterpene extracted from *Ganoderma lucidum* (Curtis) P. Karst., exhibiting antioxidant, anti-inflammatory, and anti-tumor properties (Gill and Navgeet, 2019; Jia et al., 2021; Yang et al., 2018). Pro-inflammatory cytokines (TNF-α, IL-1β, and IL-6) and the anti-inflammatory cytokine (IL-10) facilitate the progression of depressive illness (Qin et al., 2020). GAA has been shown to mitigate the inflammatory response in PSD rats by reducing the levels of TNF-α, IL-1β, and IL-6, while increasing IL-10, indicating an anti-inflammatory action of GAA. Further examination of microglial cell markers suggests that the anti-inflammatory effect of GAA in PSD rats may be mediated through the induction of the anti-inflammatory M2 phenotype in microglial cell polarization (Zhang et al., 2021).

#### 4.2.2 Regulation of NLRP3 inflammasome activity

The NLRP3 inflammasome, consisting of NLRP3, caspase-1, as well as apoptosis-associated speck-like protein, plays a crucial role in the inflammatory response by modulating the maturation of IL-1β and IL-18 (Swanson et al., 2019). The activation of the NLRP3 inflammasome may induce inflammation (Franke et al., 2021), enhance BBB permeability (Wang et al., 2020), exacerbate recurrent stroke (He et al., 2020), and the activation of hippocampal microglial NLRP3 inflammasome vesicles can facilitate chronic stress-induced depression-like behavior in rats (Feng et al., 2019).

Morinda officinalis oligosaccharides (MOOs), a herbal component derived from the roots of *Morinda officinalis* F. C. How, can tonify the kidneys and enhance immunity (Zhang J. H. et al., 2018), and regulate inflammation and apoptosis (Liang et al., 2017; Shin et al., 2013). After experimental studies, MOOs downregulated the mechanism of NLRP3 inflammasome expression in PSD rats by affecting the IκB/NF-κB p65 pathway. MOOs inhibited the activation of microglial NLRP3 inflammasomes, which attenuated hippocampal inflammation. Experimental evidence indicated that the NLRP3 inflammasome may serve as a viable target for PSD therapy, and that MOOs might alleviate depressive-like behaviors as well as hippocampal inflammation in a PSD rat model by reducing the activation of microglial NLRP3 inflammasomes (Li et al., 2021).

### 4.3 Regulating monoamine neurotransmitters

Gastrodin, the active ingredient in *G. elata* Bl., exhibits antidepressant properties (Chen et al., 2016). In assay experiments using rat microdialysate samples, three MANTs—DA, 5-HT, and L-DOPA showed significantly different dynamic concentration changes in PSD model rats. These changes were synchronized with the hematological metabolism of gastrodin (200 mg/kg) following oral administration. A SILD-MDSPE-UHPLC-MS/MS method was developed to precisely quantify trace neurotransmitters in rat brain microdialysis fluids by generating isotopically labeled m/z 413.3/416.3 and m/z 429.4/432.4 product-specific ions from d_0_- or d_3_-MRSF-labeled MANTs and aromatic amino neurotransmitters, respectively, for multiple reaction monitoring detection. The findings suggest that gastrodin has a regulatory effect on brain neurotransmitters comparable to that of fluoxetine and may serve as a promising herbal agent for the treatment of PSD (Zhao et al., 2019).

Huperzine A (HupA), an alkaloid derived from the traditional Chinese medicine *Huperzia serrata* (Thunb. ex Murray) Trevis., is a highly promising clinical drug (Wang et al., 2006). It not only inhibits acetylcholinesterase but also provides neuroprotective benefits against brain ischemic damage (Zhou et al., 2001) and improves cognitive dysfunction in major depression (Zheng et al., 2016). Following the injection of HupA, the sucrose preference of PSD rats in the sucrose preference test was significantly increased, but their resting time in the forced swimming test was dramatically decreased. HupA treatment demonstrated obvious antidepressant effects, enhancing neurological and cognitive functions in PSD rats. It was associated with the upregulation of 5-HT 1A receptor, p-CREB, as well as BDNF expression in the hippocampus, along with increased levels of 5-HT in the hippocampus and prefrontal cortex and elevated DA and NE levels in these regions. These findings indicate that HupA alleviates depressive symptoms in PSD rats, highlighting its potential antidepressant properties (Du et al., 2017).

Astragaloside VI (AsVI), a compound derived from *Astragalus membranaceus* (Fisch.) Bunge, exhibits neuroprotective effects against cerebral ischemic injury (Liu et al., 2015) and promotes axonal regeneration and neuronal synapse reconstruction (Yang et al., 2013). *In vitro*, AsVI enhances cell proliferation and migration by activating the epidermal growth factor receptor/extracellular signal-regulated kinase (EGFR/ERK) signaling pathway, while *in vivo*, it accelerates both sterile and infected wound healing (Lee et al., 2018). In PSD rats, AsⅥ treatment significantly reduced depressive-like behavior and mitigated corticosterone (CORT)-induced apoptosis in neuronal PC-12 cells. Additionally, AsVI therapy effectively prevented the decline of DA and 5-HT levels in the brains of PSD rats and in CORT-induced PC-12 cells. Moreover, AsⅥ activated the neuregulin-1-mediated mitogen-activated protein kinase (MAPK)/extracellular signal-regulated kinase (MEK/ERK) pathway, which correlated with PSD improvement. These findings indicate that AsⅥ alleviates depressive behaviors both *in vitro* and *in vivo* by increasing neurotransmitter levels and inhibiting neuronal apoptosis, highlighting its potential as a novel therapeutic option for PSD (Chen X. et al., 2022).

### 4.4 Adjusting oxidative stress

Naringin, a flavonoid found in the fruits of *Citrus reticulata* Blanco, a grapefruit species, has anti-inflammatory, anticancer, lipid-lowering, and antioxidant properties (Choi et al., 2001), as well as neuroprotective effects (Gaur et al., 2009; Kumar and Kumar, 2010). Naringin therapy significantly reduces oxidative damage by lowering malondialdehyde (MDA) and nitrite levels, restoring depleted glutathione concentrations, and enhancing the activities of catalase (CAT), superoxide dismutase (SOD), and glutathione-S-transferase (GST), indicating its antioxidant efficacy. At the cellular level, naringin alleviated the harmful effects of ROS by directly upregulating the transcription of genes encoding antioxidant enzymes, including CAT, SOD, and glutathione peroxidase (Harkin et al., 2004). Furthermore, naringin significantly restores mitochondrial enzyme activities, suggesting its role in mitochondrial function. Pretreatment with L-NAME or 7-NI further enhanced its protective effects by restoring mitochondrial enzyme activities, highlighting the involvement of nitric oxide in its neuroprotective actions. These findings suggest that naringin plays a crucial role in counteracting neurobehavioral, biochemical, and cellular changes in depressed mice following stroke (Aggarwal et al., 2010).

Rosmarinic acid (RA) is a potent polyphenol widely distributed among plant groups such as Labiatae, Seaweeds and Ferns (Petersen and Simmonds, 2003; Lee et al., 2016). RA has strong anti-inflammatory (Grigoletto et al., 2016) and antioxidant properties (Fujimoto et al., 2010; Fonteles et al., 2016). Nrf2 is a major regulator of the antioxidant signaling system. Under stress conditions, Nrf2 separates from Keap1 and translocates to the nucleus, where it initiates the transcription of antioxidant enzyme genes, thereby promoting cellular protection. Nrf2 knockdown abolished the RA-induced elevation of SOD and CAT activity, suggesting that the neuroprotective effect of RA in PSD may be compromised by the downregulation of Nrf2. These findings indicate that the Nrf2 antioxidant pathway may contribute to the antidepressant effects of RA therapy. Ischemia-reperfusion injury significantly reduced the activities of SOD and CAT, along with glutathione (GSH) levels; however, RA treatment mitigated these reductions. The protective effect of RA against ischemia-reperfusion was largely attributable to its antioxidant properties. Consequently, RA markedly reduced neurological impairments and infarct volume. RA therapy alleviated depressive behaviors by preventing the decline of SOD and CAT activities and preserving GSH levels in the brain (Wang J. et al., 2021).

Resveratrol (RES) is a polyphenolic compound and phytoantimicrobial primarily found in the skins of red wine grapes, *Reynoutria japonica* Houtt., and certain nuts. It exhibits anti-inflammatory, antioxidant, as well as anticarcinogenic properties (Gambini et al., 2015) and provides neuroprotection after stroke (Hou et al., 2018). In PSD rats, RES treatment led to reduced cytoplasmic Nrf2 expression and increased total Nrf2, heme oxygenase-1 (HO-1), and nuclear Nrf2 (N-Nrf2) protein levels, suggesting enhanced activation of the Nrf2/HO-1 pathway. These findings indicate that the suppression of the Nrf2/HO-1 pathway partially mitigated the anti-inflammatory and antioxidative effects of RES, along with its ability to enhance cognitive/learning functions in PSD rats. In contrast, activators of the Nrf2/HO-1 pathway amplified these beneficial effects. Thus, the Nrf2/HO-1 pathway may be pivotal in mediating the therapeutic benefits of RES against oxidative stress, inflammation, as well as cognitive impairment in PSD rats (Bai et al., 2024).

### 4.5 Adjustment of HPA axis

Trans-resveratrol (Trans-RES) is the main active ingredient in *R. japonica* Houtt. and is also found in dietary sources such as grapes and red wine (Bai et al., 2010). It has antioxidant, anti-inflammatory, and neuroprotective properties and has been shown to improve learning and memory impairments (Chen et al., 2007; Ranney and Petro, 2009). Cerebral ischemia affects the neuroendocrine system by activating the HPA axis, resulting in behavioral problems. Studies have demonstrated that an increased adrenal weight-to-body weight ratio is associated with adrenal hyperplasia in MCAO rats. In addition, corticotropin-releasing factor expression was elevated in the frontal cortex, hippocampus, and hypothalamus of MCAO rats. High doses of trans-RES significantly mitigated these changes, suggesting an overall improvement in neuroendocrine function. Moreover, trans-RES enhanced BDNF expression in the frontal cortex, hippocampus, and hypothalamus of MCAO rats, indicating its potential neuroprotective role in ischemic injury. These findings highlight trans-RES’s mechanism of action at the psycho-neuroendocrine level, particularly as a modulator of the HPA axis (Pang et al., 2015).

### 4.6 Regulation of the microbe-GBA

**FIGURE 5:**
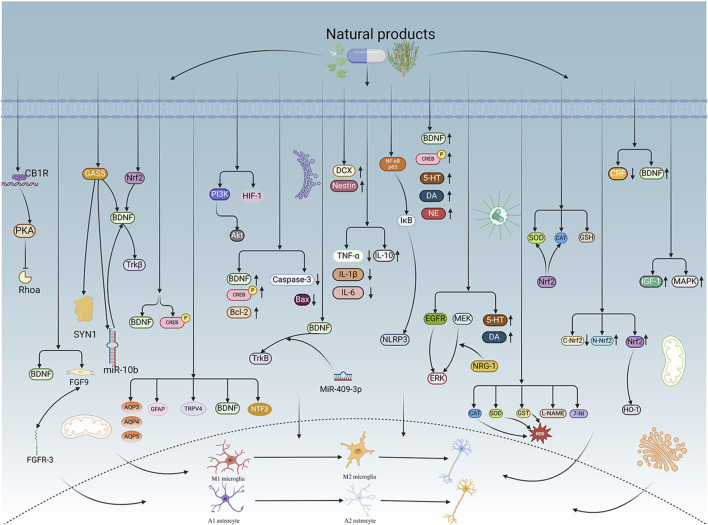
Mechanisms of action of natural products. NPs can exert antidepressant effects through relevant targets or signalling pathways, including enhancing neuroplasticity, alleviating neuroinflammation, modulating monoamine neurotransmitters, regulating oxidative stress, stabilizing the HPA axis, and maintaining the balance of the microbe-GBA. NPs further contribute to the treatment of PSD by alleviating PSD symptoms through appropriate pathological mechanisms. CB1R, Cannabinoid receptor 1; PKA, protein kinase A; Rhoa, Ras Homolog Family Member A; BDNF, brain derived neurotrophic factor; FGF9, Fibroblast growth factor 9; FGFR-3, Fibroblast Growth Factor receptor-3; GAS5, growth arrest-specific transcript 5; SYN1, synapsin 1; miR-10b, microRNA-10b; Nrf2, Nuclear factor erythroid 2-related factor 2; TrkB, Tropomyosin receptor kinase B; p-CREB, phosphorylated CREB; AQP3, Aquaporin 3; AQP4, Aquaporin 4; AQP5, Aquaporin 5; GFAP, Glial fibrillary acidic protein; TRPV4, Transient Receptor Potential Vanilloid 4; NTF3, neurotrophin-3; PI3K, phosphatidylinositol 3-kinase; Akt, V-akt murine thymoma viral oncogene homolog; HIF-1, Hypoxia-inducible factor 1; Bcl-2, B cell lymphoma-2; MiR-409-3p, MicroRNA-409-3p; DCX, doublecortin; TNF-a, Tumor Necrosis factor-Alpha; IL-1β, Interleukin-1β; IL-6, Interleukin 6; IL-10, Interleukin-10; NF-KB p65, nuclear factor-kB p65; lkB, liver kinase B; NLRP3, NOD-like receptor family pyrin domain containing 3; 5-HT, 5-hydroxytryptamine; DA, dopamine; NE, Norepinephrine; EGFR, Epidermal growth factor receptor; ERK, extracellular signal-regulated kinase; MEK, Mitogen-activated protein kinase; NRG-1, neuregulin-1; CAT, catalase; SOD, superoxide dismutase; GST, glutathione-S-transferase; ROS, reactive oxygen species; L-NAME, L-arginine methyl ester; 7-NI, 7-nitro-indazole; GSH, glutathione; C-Nrf2, C-Nuclear factor erythroid 2-related factor 2; N-Nrf2, N-Nuclear factor erythroid 2-related factor 2; HO-1, heme oxygenase-1; CRF, corticotropin-releasing factor; IGF-1, insulin-like growth factor 1; MAPK, mitogen-activated protein kinases. Created in BioRender. Gao, H. (2025) https://BioRender.com/8hwtv6t.

Inulin is a natural intestinal regulator that balances GM, corrects endocrine dysfunction, and enhances immune responses (Vandeputte et al., 2017; Wan et al., 2020). Its consumption promotes the proliferation of beneficial gut bacteria, which are linked to the production of insulin-like growth factor 1 (IGF-1), an endocrine hormone that reduces body weight by regulating fatty acid synthesis and lipid metabolism (Birkeland et al., 2020; Le Bastard et al., 2020). Inulin crosses the BBB and influences neuronal development, proliferation, and differentiation via the MAPK signaling pathway (Yang et al., 2021). In PSD rats, inulin consumption significantly enhanced MAPK signaling in the hippocampus and altered GM composition, increasing *Lactobacillus* and *Clostridium* populations while reducing body weight. Additionally, inulin supplementation elevated IGF-1 levels in the bloodstream, activating the MAPK pathway and alleviating depression-like behaviors in PSD rats (Shao et al., 2024) (Figure 5).

## 5 Toxicology and side effects

Although NPs offer therapeutic potential for PSD through certain mechanisms, they also present toxicological risks and side effects. The simultaneous presence of pharmacological efficacy and toxicity necessitates careful evaluation, as some adverse effects may outweigh their therapeutic benefits (Guo et al., 2024). For example, Curcumin has a maximum tolerated dose of 8 g/day in clinical trials, with higher doses leading to dose-limiting toxicity, including neutropenia, anemia, and severe diarrhea. Additional side effects such as oral mucositis, hand-foot syndrome, nail and skin changes, conjunctivitis, and fatigue have also been reported (Bayet-Robert et al., 2010). Moreover, the clinical application of many natural compounds is limited by their low water solubility, poor oral bioavailability, and limited chemical stability (Nagahama et al., 2016; Zhang et al., 2023).

AE has been shown to exhibit significant hepatotoxicity and nephrotoxicity. The methanolic extract of *Cassia occidentalis* seeds demonstrated significant cytotoxicity against primary rat hepatocytes and HepG2 cells, suggesting that AE may be a major toxic component (Panigrahi et al., 2015). In addition, AE induced primary DNA damage in the liver and kidney, as shown in an *in vivo* mice comet experiment (Nesslany et al., 2009), further supporting its toxic effects on these organs. AE exposure combined with ultraviolet radiation also caused significant phototoxicity in human skin fibroblasts. The results suggest that a photochemical mechanism involving singlet oxygen species is the most probable pathway responsible for the observed phototoxicity (Vath et al., 2002).

SSA has therapeutic potential for various diseases at appropriate dosages; however, excessive intake may lead to acute or cumulative chronic hepatotoxicity and gastrointestinal complications (Bermejo et al., 2002; Wang et al., 2010). SSA induces oxidative stress in a time- and dose-dependent manner, leading to hepatic damage, as shown by increased serum aminotransferase activity, elevated hepatic SOD activity, and upregulated MDA levels in both mice and rats (Lv et al., 2009; Huang and Sun, 2010). A proteomic study investigating SSA-induced liver injury identified 487 differentially expressed proteins, with several upregulated proteins associated with lipid metabolism, protein metabolism, macromolecular transport, cytoskeletal structure, and stress response (Li et al., 2017). Acute liver damage in rats was only observed at SSA doses exceeding 12.957 mg/kg, which is eight times higher than the clinically safe dosage, indicating that the pharmacological and toxicological effects of SSA are dose-dependent. Meanwhile, comparative toxicological studies revealed a significant overlap between the proteins modified by SSA in acute liver and kidney injuries, suggesting a potential risk of nephrotoxicity at elevated dosages of SSA (Li et al., 2018).

Limited toxicological studies have been conducted on gastrodin; however, clinical reports indicate adverse drug reactions, primarily affecting the skin, gastrointestinal tract, and nervous system. Intramuscular injection of gastrodin can cause severe allergic reactions and even anaphylaxis (Zheng et al., 2015).

AsVI has several pharmacokinetic limitations, including poor intestinal permeability, high molecular weight, low lipophilicity, and dependence on paracellular transport (Huang et al., 2006). Preclinical toxicity evaluations indicate that intravenous administration of AsVI at 1.0 mg/kg in rats induces maternal toxicity, while doses exceeding 0.5 mg/kg exhibit fetotoxic effects without teratogenicity (Jiangbo et al., 2009). Further reproductive toxicity studies in rats revealed that maternal exposure to 1.0 mg/kg AsⅥ for 4 weeks led to developmental delays in offspring, including delayed fur development, delayed eye opening, and impaired cliff avoidance reflexes (Xuying et al., 2010).

In toxicity studies, carnosic acid caused histopathological changes in the heart, liver and kidneys (Wang et al., 2012). STS induces cell death and reduces cell viability in a dose-dependent manner (Tan et al., 2014). Toxicity tests have shown that curculigoside is toxic and may cause cold sweats and numbness of the limbs, as well as liver damage (Jiao et al., 2013). MOOs may have adverse reactions such as irritability, insomnia and malaise if overdosed (Qiu et al., 2016). HupA has mild to moderate cholinergic side effects and even adverse reactions such as muscle tremors, drooling, tearing, and increased bronchial secretions (Ha et al., 2011). Studies have found that resveratrol may cause diarrhoea, nausea, vomiting, flatulence, abdominal cramps, headache, and rash, and even dose-dependent gastrointestinal side effects (La Porte et al., 2010).

In summary, NPs have shown clinical efficacy in various studies and trials. However, their potential toxicity and side effects should not be overlooked. The toxicological issues associated with these compounds are often complex and diverse, with different active ingredients triggering distinct adverse reactions. Some toxicities, such as allergic and gastrointestinal reactions, may manifest shortly after administration, whereas others, like chronic liver or kidney damage, may emerge only after prolonged exposure. These toxic or side effects can significantly impact human health, ranging from mild cases that reduce quality of life to severe, life-threatening conditions. Therefore, thorough toxicity evaluations of NPs is an important task that cannot be ignored. Future research should focus on elucidating their toxicological mechanisms to ensure a clearer understanding of their safety and potential risks, ultimately providing a more scientific and reliable basis for their safe clinical application (Table 2).

**TABLE 2:** Dosage for toxicological research and side effects of natural products.

Natural product	Dosage for toxicological research	Side effects	References
Gastrodin	50 mg/kg, 100 mg/kg, 200 mg/kg	Adverse reactions to gastrodin occur mainly in the skin, gastrointestinal tract and nervous system, and studies have shown that intramuscular injection of gastrodin can cause severe allergic reactions and even anaphylaxis.	Wang et al. (2025), Zheng et al. (2015)
Carnosic acid	7,100 mg/kg	In toxicity studies, carnosic acid caused histopathological changes in the heart, liver and kidneys.	Wang et al. (2012)
Curcumin	8 g/day	Curcumin has dose-limiting toxicity (neutropenia, anaemia and severe diarrhoea) and may also cause oral mucositis, hand-foot syndrome, nail changes, skin changes, conjunctivitis and fatigue.	Bayet-Robert et al. (2010)
Aloe-Emodin	500 mg/kg, 1,000 mg/kg, 2000 mg/kg	AE can cause primary DNA damage in the liver and kidney. Significant phototoxicity was also induced in human skin fibroblasts when exposed to AE and UV radiation.	Nesslany et al. (2009), Vath et al. (2002)
Sodium Tanshinone IIA Sulfonate	320 μmol/L	STS induces cell death and reduces cell viability in a dose-dependent manner.	Tan et al. (2014)
Saikosaponin A	12.957 mg/kg	SSA overdose can cause acute or accumulation-related chronic hepatotoxicity and other digestive disorders. There is also a potential risk of nephrotoxicity with high doses of SSA.	Bermejo et al. (2002), Wang et al. (2010), Li et al. (2018)
Curculigoside	3–9 g/day	Toxicity tests have shown that curculigoside is toxic and may cause cold sweats and numbness of the limbs, as well as liver damage.	Jiao et al. (2013)
Morinda officinalis oligosaccharides	1,000 mg/kg	MOOs may have adverse reactions such as irritability, insomnia and malaise if overdosed.	Qiu et al. (2016)
Huperzine A	0.1 mg/kg/d, 0.6 mg/kg/d	HupA has mild to moderate cholinergic side effects and even adverse reactions such as muscle tremors, drooling, tearing, and increased bronchial secretions.	Ha et al. (2011)
Astragaloside VI	1.0 mg/kg, 0.5 mg/kg	AsⅥ has been found to be maternally and foetotoxic in toxicity tests and causes problems with fur development and delayed eye opening and cliff avoidance reflexes.	Xuying et al. (2010)
Resveratrol	0.5 g/day	Studies have found that resveratrol may cause diarrhoea, nausea, vomiting, flatulence, abdominal cramps, headache, and rash, and even dose-dependent gastrointestinal side effects.	La Porte et al. (2010)

AE, Aloe-Emodin; DNA, DeoxyriboNucleic Acid; UV, Ultraviolet; STS, Sodium Tanshinone IIA Sulfonate; SSA, Saikosaponin A; MOOs, Morinda officinalis oligosaccharides; HupA, Huperzine A; AsVI, Astragaloside VI.

## 6 Conclusions and prospects

PSD is a common neuropsychiatric disorder that has garnered increasing attention due to the growing emphasis on mental and emotional wellbeing alongside physical health. As research on NPs expands, their potential in the treatment of PSD has become an area of growing interest. Current studies suggest that NPs exert antidepressant effects through multiple mechanisms, including enhancing neuroplasticity, alleviating neuroinflammation, modulating monoamine neurotransmitters, regulating oxidative stress, stabilizing the HPA axis, and maintaining the balance of the microbe-GBA.

Although NPs hold significant research value in the treatment of PSD, most studies are currently based on animal models, with limited clinical validation. This lack of clinical evidence makes it difficult to accurately assess their therapeutic efficacy in patients. Additionally, despite demonstrated antidepressant effects, NPs face challenges such as low water solubility, poor intestinal permeability, limited oral bioavailability, and potential liver and kidney toxicity. Their pharmacological effects are also influenced by time- and dose-dependent factors, which may hinder clinical application and therapeutic effects. Furthermore, most studies focus on a single target or a specific signaling pathway, with limited exploration of the interactions between different targets and pathways.

In future scientific research, comprehensive and extensive clinical trials should be carried out to further investigate and evaluate the therapeutic effects of NPs on PSD. At the same time, research on the toxicology and side effects of NPs should be strengthened to minimize toxicity, reduce the toxicity and side effects, and expand their application, thereby providing patients with safe and reliable treatments. Additionally, both the horizontal and vertical research should be enhanced by integrating cutting-edge technologies such as spatial multi-omics and single-cell sequencing to bring more effective and safe intervention strategies for patients with PSD. In addition, these advanced technologies should be utilized to explore the interaction mechanisms between multiple targets and signaling pathways, ultimately improving therapeutic approaches for PSD.
